# Complex Stability and an Irrevertible Transition Reverted by Peptide and Fibroblasts in a Dynamic Model of Innate Immunity

**DOI:** 10.3389/fimmu.2019.03091

**Published:** 2020-02-14

**Authors:** Abulikemu Abudukelimu, Matteo Barberis, Frank Redegeld, Nilgun Sahin, Raju P. Sharma, Hans V. Westerhoff

**Affiliations:** ^1^Synthetic Systems Biology and Nuclear Organization, Swammerdam Institute for Life Sciences, University of Amsterdam, Amsterdam, Netherlands; ^2^Molecular Cell Physiology, VU University Amsterdam, Amsterdam, Netherlands; ^3^Systems Biology, School of Biosciences and Medicine, Faculty of Health and Medical Sciences, University of Surrey, Guildford, United Kingdom; ^4^Centre for Mathematical and Computational Biology, CMCB, University of Surrey, Guildford, United Kingdom; ^5^Division of Pharmacology, Department of Pharmaceutical Sciences, Faculty of Science, Utrecht University, Utrecht, Netherlands; ^6^School for Chemical Engineering and Analytical Science, University of Manchester, Manchester, United Kingdom; ^7^Systems Biology Amsterdam, VU University Amsterdam, Amsterdam, Netherlands

**Keywords:** innate immunity, inflammation, irreversible transitions, bi-stability, fibroblasts, systems biology

## Abstract

We here apply a control analysis and various types of stability analysis to an *in silico* model of innate immunity that addresses the management of inflammation by a therapeutic peptide. Motivation is the observation, both *in silico* and in experiments, that this therapy is not robust. Our modeling results demonstrate how (1) the biological phenomena of acute and chronic modes of inflammation may reflect an inherently complex bistability with an irrevertible flip between the two modes, (2) the chronic mode of the model has stable, sometimes unique, steady states, while its acute-mode steady states are stable but not unique, (3) as witnessed by TNF levels, acute inflammation is controlled by multiple processes, whereas its chronic-mode inflammation is only controlled by TNF synthesis and washout, (4) only when the antigen load is close to the acute mode's flipping point, many processes impact very strongly on cells and cytokines, (5) there is no antigen exposure level below which reduction of the antigen load alone initiates a flip back to the acute mode, and (6) adding healthy fibroblasts makes the transition from acute to chronic inflammation revertible, although (7) there is a window of antigen load where such a therapy cannot be effective. This suggests that triple therapies may be essential to overcome chronic inflammation. These may comprise (1) anti-immunoglobulin light chain peptides, (2) a temporarily reduced antigen load, and (3a) fibroblast repopulation or (3b) stem cell strategies.

## Introduction

When activated leukocytes engage in host defense against an invading antigen, the process of innate immunity often reverts disease progression within a fortnight (1, 2). Occasionally, this acute and transient inflammation turns into a chronic inflammation with local tissue damage (3–5). The chronic inflammation consists first of an accumulation phase, which is characterized by rapid recruitment of immune cells (i.e., macrophages, eosinophils, neutrophils, mast cells). This is followed by the activation phase, where various cytokines and chemokines are released by immune cells. Then, a subversion phase follows, when the chronic inflammation subsides. Chronic inflammation can last many months and even years, continuing even in the absence of the invading foreign antigen. This lengthy period can cause much progressive damage to normal tissues and thereby invoke numerous functional, hence financial and societal, complications, affecting the patient, the family, and the healthcare system (6–8). Meanwhile, the slower but more specific adaptive immunity process will begin, but this is beyond the scope of this paper and its mathematical model.

In many studies of immunity, *in silico* modeling has been used. One modeling avenue has aided a structure-based drug design, focusing on single molecules and their interactions, for instance, identifying the antagonists of CCR4 *in silico*, validated by injection together with vaccines *in vivo* (9). Another branch of modeling focuses on the networks within any one particular cell that are of immunological importance. This comes close to more conventional systems biology (10). The present paper treads a third avenue, i.e., models of the dynamics of the intercellular networks in immunology. Mathematical models based on ordinary differential equations (ODEs) have already been used to study B cell responses (11, 12), T cell responses (13, 14), and natural killer (NK) cell mechanisms (15, 16), but largely in adaptive immunity. Various software environments already exist for modeling immune system paradigms (17). For example, the immune cell simulator (18), the synthetic immune system (19), and the basic immune simulator (20) provide platforms for creating and then simulating the performance of virtual immune systems consisting of a variety of cell types and their interactions. These models and environments have not yet championed the systems biology approach of examining how molecular and (in this case) cellular interaction mechanisms lead to the functional properties of the network, of comprehensive maps of the interaction network, of systematic notation of such maps, of allowing the implementation of genomics information, and of dynamic models directly exchangeable through SBML nor have they connected systems biology methodologies such as control analysis with its summation laws.

Such systems biology strategies, which typically integrate quantitative experimental information with such network modeling, may help to construct network–mechanistic understanding of the immune cells' roles (21), especially if the models consist of realistic processes. On the other hand, immunology may also help in the development of multicellular systems biology. Immune cells which are involved in host defense can be isolated from living organisms, cultured *in vitro*, and reconstituted into another living organism *in vivo*. Consequently, innate immunity may be one of the few areas in human biology where complex system properties arising through “multicellular system emergence” can be identified and understood in an integral experimental and computational analysis. Such emergent properties are likely to include the maintenance of phenotypic stability in the face of perturbations caused by infection and inflammation, resulting from both positive and negative feedback loops that together accomplish a strong yet limited response, but they should also include deviations from such stability, which may then resemble human pathologies such as chronic inflammation and allergy.

Although from the perspective of understanding the immune response, innate immunity has already been proposed a while ago to be most suitable for systems biology studies (10), the number of mathematical models focusing on this innate immunity is still limited (22), yet the innate immune system plays an important role in effective host defense against bacterial and viral infections (23, 24) and possibly in allergies and tumorigenesis, and there may already be sufficient information to construct explicit mechanism-based systems biology models. A comprehensive map of IgE-mediated mast cell activation (25) might be used to explore both the intracellular and population dynamical properties of the corresponding section of the immune system, and the experimental field is now reporting genome-wide experimental data sets, which systems biology can integrate and help understand in terms of the emergence of function (10). One pipeline of already existing mathematical models of innate immunity has only four, rather abstract, variables, i.e., “inflammation,” “damage,” “initiating event,” and “anti-inflammation” (26). This pipeline already shows that complex functional behavior emerges at certain values of the abstract parameters. These models are simplifications of a much more detailed and explicitly mechanistic model that enabled *in silico* simulation of clinical trials of anti-TNF (TNF = TNF-α, i.e., tumor necrosis factor alpha) agents as medicine against sepsis (27). The simulations helped explain why so many of these clinical trials have failed (28): inter-patient variability and the role of multiple factors rather than of the single drug target (27). More recently, Petersen et al. (29) put in place a deep reinforcement learning strategy, automatically personalizing this type of model for use in multifold cytokine therapies. Mavroudis et al. (30) showed how stochasticity can be taken into account, which they did essentially for the simpler four-variable model referred to above. Other authors extended these models to include spatial dimensions and multiscale aspects (22, 31), the latter authors shifting to a Boolean approach.

The models have already been used in the context of applications to actual diseases and their therapies. Prince et al. (32) combined an *in silico* endeavor with an *in vivo* approach to elucidate the complexity of inflammation in CD-14-deficient mice subjected to Gram-negative lipopolysaccharides and cannulation. Rullmann et al. developed the Entelos Rheumatoid Arthritis Physiolab platform to predict the therapeutic effects of drugs (33). Separately, the National Institute of Allergy and Infectious Diseases established the Systems Biology for Infectious Diseases Research (SysBio) program that facilitates research lines in systems influenza, systems virology, systems biology of enteropathogens, and *Mycobacterium tuberculosis* systems biology (34), and there is much application in the realm of tumorigenesis [e.g., (35–37)].

We became interested in applications with respect to the curing of inflammation using peptides interfering with extracellular regulatory pathways (34). This came about after realizing that immunoglobulin light chains may help elicit inflammation (38) and occur at increased levels in inflammatory pathologies (39). Anti-FLC peptide F991, a functional inhibitor of the immunoglobulin light chain either in its free form or when in IgE (40), appeared to be able to slow down the progression of colitis, presumably by reducing inflammation (41), but in our own hands, the results were variable. To try to understand both the effect and its complexity/variability, we constructed a detailed differential equation model (42). This model enacted inflammation as a set of explicit positive and negative regulatory loops involving host tissue (fibroblasts), B cells, mast cells, and microbial cells, as well as TNF, circulating antigen, IgE receptors on the mast cells, and immunoglobulin light chains, free or in igE. As detailed in Abudukelimu (42), the model was based on well-known interactions in innate immunity, on reasonable physical chemical parameter values (inclusive of consistency with diffusion limitation and equilibrium binding constants), and validated in the sense of predicting known features of innate immunity. In making one of these predictions, we plotted the model-predicted TNF level as marker of the intensity of inflammation vs. antigen dosage. We observed a sustained low level of inflammation at low antigen dosage, until at a certain antigen dosage the TNF flipped to a much higher level. Bringing the antigen dosage back down did not bring the TNF level down. Only at a very low antigen dosage did the TNF lead to low inflammation levels. Then, two surprises emerged: first, a minor increase in antigen dosage already pushed the TNF back to high inflammation levels and, second, simulated F991 peptide therapy worked only transiently. It seemed that the model behaved non-robustly when changing the antigen dosage or when treating with the peptide. In the earlier paper (42), we noted these phenomena and suggested that bistability might be at the origin of this, but we did not analyze and prove this further. When subsequently we began to analyze the phenomenon in more detail, we found that it was not just a simple bistability but much more complex than that, the complications having potential impact on innate immunity, allergies, and therapies thereof. It is these phenomena that we shall embark on here with more targeted systems biology methodologies. Non-unique behavior of non-linear models has been understood by stability and bifurcation analysis, e.g., in epidemiology, physiology, and immunology (26, 43). Such analyses can reveal that a model predicts multiple, stable or metastable, steady, and oscillatory or chaotic attractor “states.” Parameter sensitivity analysis can then be used to establish how readily the network function becomes critical as a parameter is changed. In the context of immunology, sensitivity analysis has, for instance, been used to evaluate the influence of maternal adaptive immunity on the time dependence of infection and on its consequences for serology (44) to identify the relative importance of the molecular components in coupled MAPK and PI3K signal transduction pathways (45), for the NF-κB signaling network (46), in an immune-based model of *Helicobacter pylori* infection (47), and in analyzing the T cell response to antigen (48). An approach related to sensitivity analysis is metabolic control analysis, which is limited to sensitivities with respect to process activities (49–51). This comes with the advantage that, for stationary states, control coefficients sum up to fixed totals of 1, 0, and −1 for control of concentrations, fluxes, and cycle times, respectively (49, 51). This helps to organize questions such as whether the total control of network performance resides in a single “rate-limiting” step or is distributed over many processes in the network. If the latter case upholds, this begs the question which components have the highest control coefficients and thereby surface as the better targets for therapeutic interventions (50).

Where the computations in our previous paper (42) observed some sort of complex bistability in the modeled innate immunity, they did not elucidate what type of complexity it was and how it could be controlled. In this paper, we implemented stability, bifurcation, and control analysis to that model of innate immunity (42) with the aim of clarifying (1) why and how it exhibits its complex dependence on antigen dosage as well as on the history thereof, (2) why the F991 peptide may act erratically, (3) if there might be a way to increase the robustness of this peptide therapy, and (4) which should be the best targets for intervention. We report on a subtle balance between apparent stability and bistability, a threshold between acute and chronic inflammation, fibroblasts playing a special role in preventing or reverting an irrevertible transition to chronic inflammation, and a wide distribution of control implying multiple potential drug targets.

## Methods

### The Dynamic Model

The dynamic model used was defined and illustrated in Abudukelimu et al. (42). Its node and balance equations, leading to ODEs, were encoded in Copasi software (52). Precisely the same equations and parameter values were used in the present paper (with minor exceptions for TNF and MMP7, as documented in Supplementary Table S1). The model's Copasi and SBML code are in the Supplementary Material of this paper and in the JWS-Online model repository. The model has B cells, mast cells, fibroblasts, and bacteria as cellular components and cross-reactive antigen (CRA), protease, tumor necrosis factor-α (TNF), MMP7, MMP8, free light chains (FLCs or IgE) and their receptors on mast cells, and FLC-binding peptides as molecular components. There is an influx of CRA and, where indicated, an influx of fibroblasts or presence of bacteria. Molecular components, fibroblasts, and (if present) bacteria wash out at a certain rate but are also produced by the biology. The parameter “total space” is 1,000 fM, such that fibroblasts could only grow to a concentration of 1,000 fM. This corresponds to 1.7 pl per cell, which is when they should occupy “total space” and be contact-inhibited in three dimensions.

Units in the model are as indicated, typically picomolar (pM, 10^−12^ moles/liter), femtomolar (fM, 10^−15^ moles/liter), or attomolar (aM, 10^−18^ moles/liter). The results being reported in terms of these real (rather than “arbitrary”) units should not be taken to suggest precision. Due to the uncertainty in parameter estimates, our results are unlikely to be precisely correct, yet expressing the results in this manner allows for a better comparison between them.

### Sensitivity and Control Analysis

Control coefficients (a type of sensitivity coefficients or response coefficients) (51) were calculated for levels of model components such as TNF at steady state considering their control by process–rate parameters such as the influx rate of CRA. They were defined as the log–log derivatives of concentration with respect to rate constant (53) and computed numerically through Copasi. They are represented as a capital C followed by the controlled variable as superscript and by the controlling parameter as subscript.

### Stability Analysis of Steady States

In order to determine the stability of the system, numerical simulations were performed by using Copasi in two modes. In one we asked it to compute the steady state as well as the stability properties at steady state in terms of the eigenvalues. When any eigenvalue was reported by Copasi to have a positive real part, we report that the state is unstable. Every steady-state computation was followed by a calculation of time dependence starting from that steady state as initial condition. Only if that second computation confirmed that the system did not evolve away from that initial condition do we report the state as stable. Depending on conditions, the computations produced one or two stable steady states. In the latter case, there often appeared to be a third unstable steady state, which Copasi could find in its steady-state mode but not in its time-dependence mode.

We did not perform a complete scan of initial conditions space nor of the parameter space since it is impossible to obtain the ultimately necessary resolution when there is such high dimensionality. We therefore cannot exclude that there are more than three steady states in the model for any given value of CRA influx rates.

### The Model and a Caveat

This paper will use our model of innate immunity (42), which constitutes an attempt to be realistic in terms of elemental mechanisms and parameter values. Parameter values and kinetic equations are not phenomenological but refer to what is or could be true, or at least possible, e.g., in the sense of diffusion limitation, as rationalized in Abudukelimu et al. (42).

### Figures

Computations were performed for many parameter values, and the results are mostly shown as very small dots, which are then connected by more visible straight lines. The points on the connecting lines have not been computed but interpolated.

## Results

### Instability, Flares of Infection, and a Therapy Thereof

**Figure 1:**
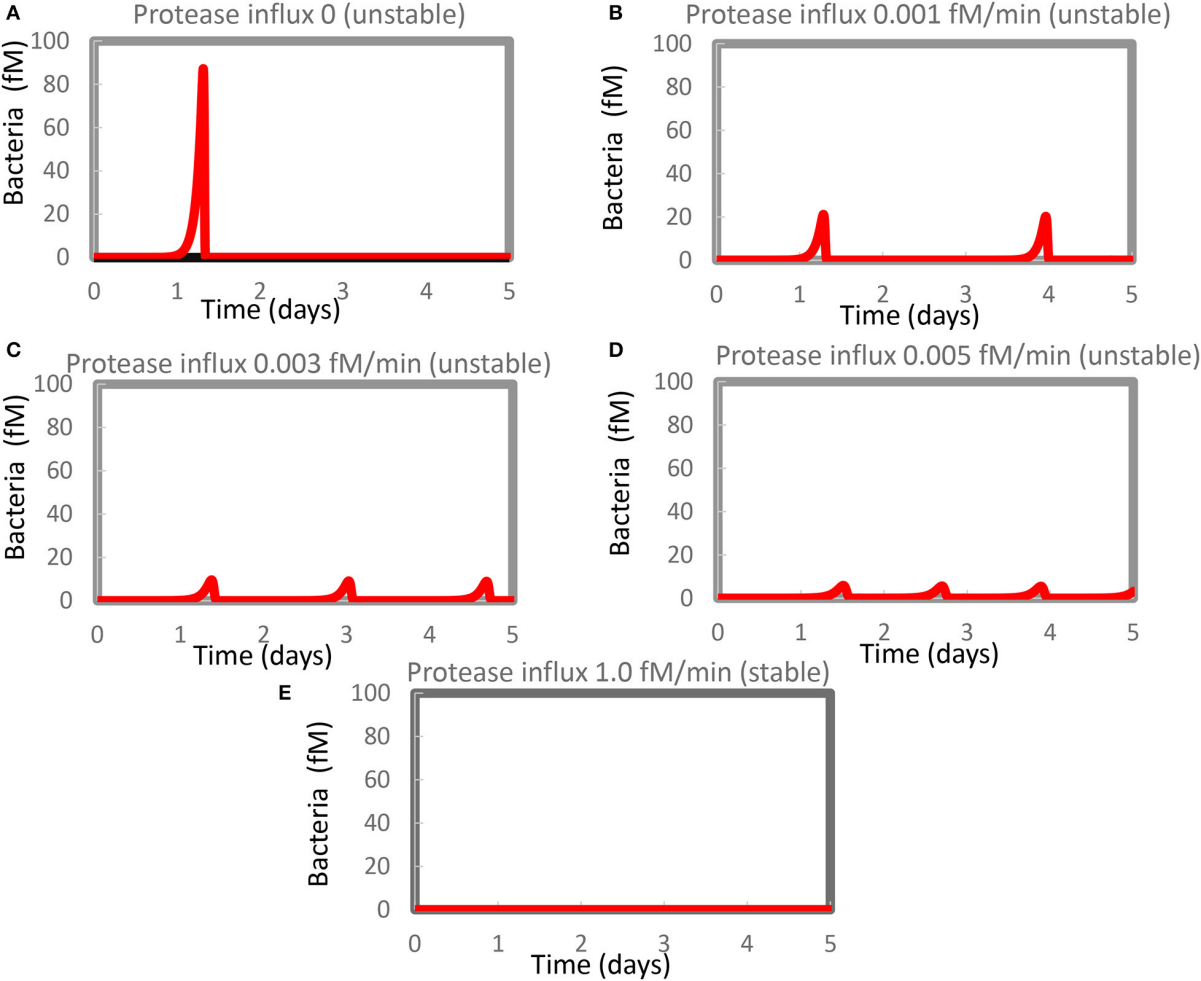
Resurgence of bacterial infection in the *in silico* model of innate immunity. The standard model (Supplementary Table S1) was re-run at various magnitudes of the steady influx of a protease that could kill the bacteria. Protease influx was **(A)** zero, **(B)** 0.001 fM/min, **(C)** 0.003 fM/min, **(D)** 0.005 fM/minm, or **(E)** 1.0 fM/min. Only for **(E)** was a stable steady state reported, with the largest eigenvalue equaling −0.0005/min. For the other four cases, unstable steady states at zero levels of bacteria were computed with positive real parts of one or (if complex) two eigenvalues. Supplementary Table S2 shows concentrations computed at 4 days and at steady state.

When added to our model of innate immunity (42), infecting bacteria grew quasi-exponentially until they were overrun by an even faster-growing immune response causing a virtually complete elimination of bacterial cell count in what seemed to be a steady state (Figure 1A) (42). In the stability analysis of the present study, we found that that final state was *not* a stable steady state however (Figure 1A); the steady state had one real eigenvalue that was positive (0.005/min). This resulted in spurious resurgence of the infection in the *in silico* model, which only became evident when we ran the model for appreciable duration. Bursts of bacterial growth then arose long after the infection appeared to have been dealt with by the innate immune system (results not shown).

A similar phenomenon has been reported by Kumar et al. (54) but attributed to computational problems. We therefore evidenced that such computational problems do not seem to be responsible for our results. For Figure 1D, for instance, the minimum obtained in between the first and the second peak amounted to 0.0007 fM of bacteria (about 0.4 million per liter), i.e., not at all close to other small numbers in some of our other robust computations (see below) which were 10^−50^ fM or smaller. This number (0.0007 fM) did not change when we varied the step size or tolerance settings of Copasi. Moreover, the steady-state algorithm led to an unstable steady state (at zero bacteria), with a complex eigenvalue with positive real part of 0.0018/min.

We therefore reckon that the phenomena of recurrent bursts of infection could be realistic and reflect the difficulty of the innate immune system in eliminating the very last bacteria. In the model, the bacteria were lysed by proteases produced by activated mast cells. This activation was part of a positive feedback loop and should vanish upon the virtual disappearance of the bacteria. This produced the paradoxical situation that the regulation that had been eliminating the bacteria disappeared when the density of the bacteria became low, enabling the bacterial population to re-emerge.

We expected that a steady influx of protease killing the bacteria (in addition to the protease produced by activated mast cells) should reduce the maximum level of infection and get rid of it more quickly and more definitively. Figures 1B–D show that our expectations were only partly correct: the amplitude of the bacterial infection was reduced by the extra protease, as expected, but now multiple bursts of bacterial growth occurred within the time span considered at a frequency that increased rather than decreased with the magnitude of the protease influx. The highest influx rate of protease that we tested *was* detrimental to the bacteria and produced a steady state without recurrence (Figure 1E). At this protease influx rate, the steady-state solution only had eigenvalues with negative real parts (the largest was −0.0005/min). A strong steady influx of protease appeared to be a “therapy” of the flares of inflammation.

### Bistability and Bimodal Control

Having identified bacterial growth as a major source of instability in our model, we wondered if there were more such sources. We therefore removed the bacteria from our computations and replaced them with a constant influx of antigen, which we call cross-reactive antigen. In our model, this CRA was already secreted by fibroblasts that are activated by TNF.

We then increased this CRA influx in steps, starting from zero. Up to a CRA influx rate of 16.7 fM/min, the inflammation intensity, as evident from TNF levels, increased only slightly with increasing CRA challenge (42). At just over 16.7 fM/min, the inflammation suddenly intensified, as witnessed by a strong increase in TNF levels and a strong decrease in fibroblast levels. When the CRA influx rate was reduced subsequently, the system stayed in the high inflammatory mode and only returned to states with low TNF levels at very low CRA challenges (see also Figure 4B below). At any CRA influx rate below 16.7 fM/min, the system could exist in either of two stable steady states (42), an apparent case of dynamic bistability (55, 56). We refer to these two families of steady states as two “modes” of inflammation, i.e., the “chronic” mode and the “acute” mode (42), for the multiple reasons that we shall detail below.

**Figure 2:**
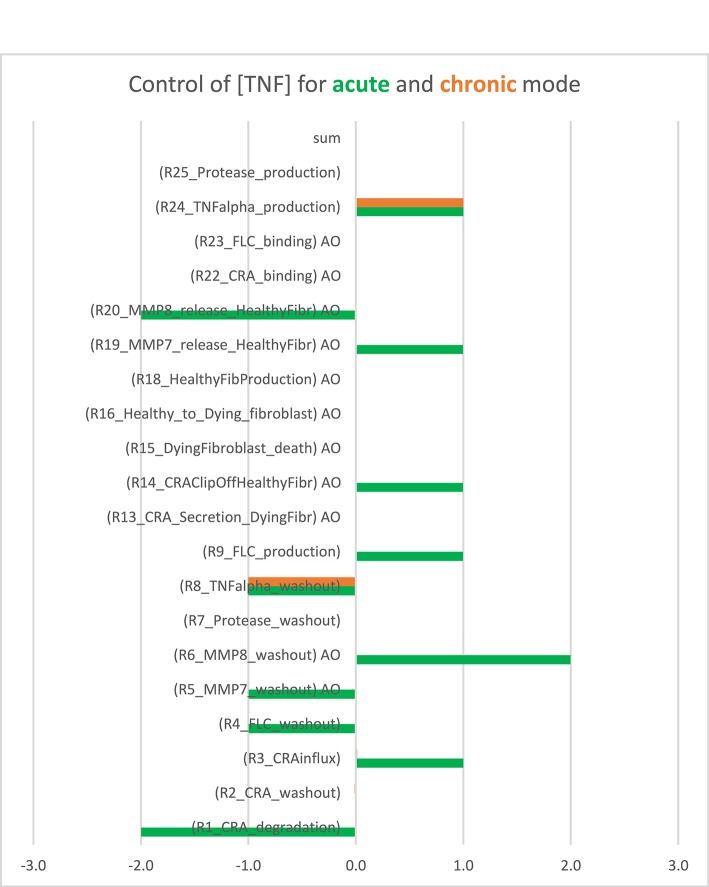
Bimodal control analysis: distribution of control of acute and chronic inflammation. Concentration control coefficients for TNF were calculated for the acute and chronic mode of inflammation and indicated as blue and red bars, respectively, with their magnitudes corresponding to their projections onto the abscissa. The standard model was used at CRA influx = 1.0 fM/min (see Supplementary Table S3). In the chronic mode, the concentration control coefficients for kinetic activities annotated by “AO” were not computable because the controlling parameter was a property of a species present at a concentration too close to zero. These control coefficients correspond to zero. Also in the other cases where no bar is visible (including the sum case) the corresponding control coefficient equals 0.0. This Figure was created using both Supplementary Table S3 (the concentration control coefficients for acute) and Supplementary Table S4 (the concentration control coefficients for chronic cases).

For each of these modes, we wondered whether inflammation is controlled by a single “rate-limiting” process, such as the influx of antigen, and if so, whether this controlling process differed between acute and chronic inflammation. To answer these questions, we performed a control analysis of both the acute and the chronic states at an intermediary antigen load of 1 fM CRA/min. For the acute mode of inflammation, control was distributed over many but certainly not all of the participating processes. As expected, there was full positive control of TNF levels by TNF production and full negative control by TNF washout, but also many other processes exercised control over the inflammation's TNF level (Figure 2, the green bars). This included positive control by release of the pro-inflammatory cytokine MMP7 by fibroblasts and an even stronger negative control by MMP8 released by the fibroblasts, as well as substantial control by CRA clip off from fibroblasts, by FLC production by B cells, by CRA influx and degradation, and by the washout of various factors. The absence of control by the fibroblast life cycle is understandable on the basis of the phenomenon that fibroblast levels in acute inflammation are pretty close to their maximum defined by contact inhibition (42). By contrast, in the chronic inflammation mode, all control resided in the production by mast cells of TNF and in the washout of TNF (orange bars in Figure 2). This was because all mast cells were already fully active in TNF production (not shown).

**Figure 3:**
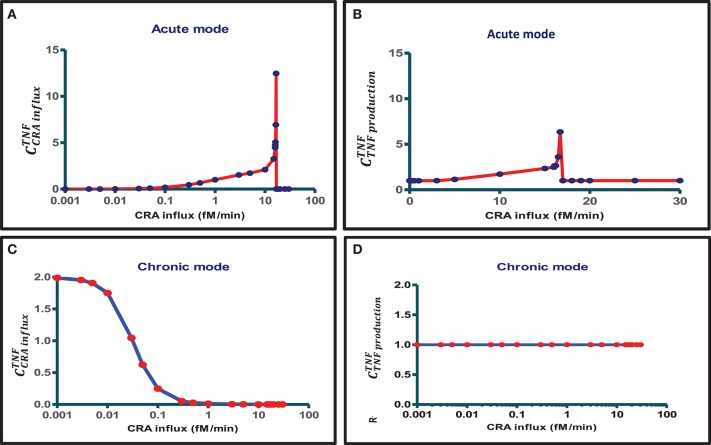
Bimodal control analysis of the innate inflammation model at various antigen challenges. A first mode of concentration control coefficients of steady-state magnitudes of TNF with respect to **(A)** CRA influx rate and **(B)** TNF production rate constant was obtained when starting from a steady state at a very low CRA influx rate (0.001 fM/min) and then increasing the CRA influx to the level indicated on the abscissa (this procedure defines the “acute inflammation mode” of the model; cf. Figure 4A). A second mode of control coefficients of steady-state magnitudes of TNF with respect to **(C)** CRA influx rate [see also (42)] and **(D)** TNF production rate constant was obtained when starting from a steady state at high CRA influx rate (30 fM/min), then decreasing the CRA influx to the level indicated on the abscissa (this procedure defines the “chronic inflammation branch” of the model; cf. Figure 4B). Control coefficients were calculated for the CRA influx value corresponding to each red point. The blue straight lines connect these computed points.

**Figure 4:**
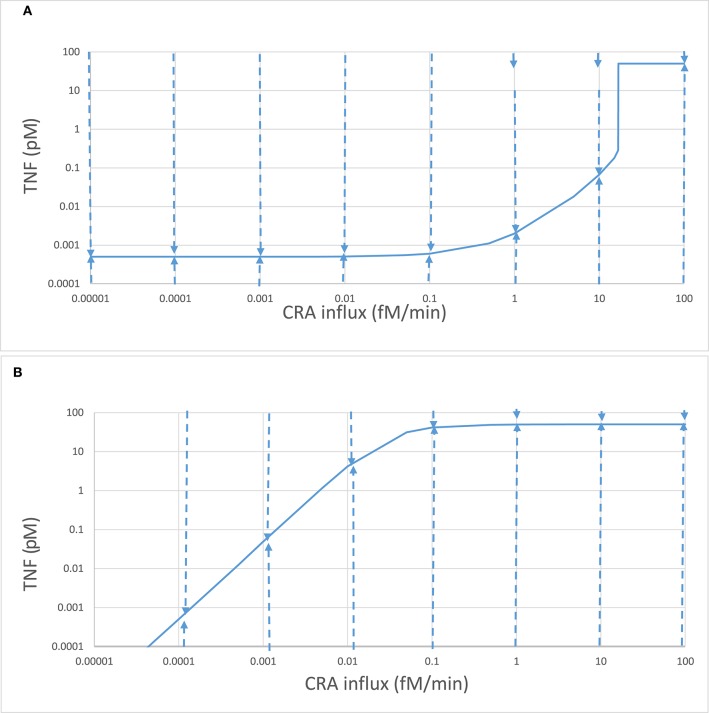
Complex stability properties of the innate inflammation model. Stepwise computations again using the model of Abudukelimu et al. (42), starting from the 0.001 fM/min CRA influx steady state for the acute mode and step-wise increasing that influx or starting from the 30 fM/min CRA influx steady state for the chronic mode and stepwise decreasing that influx. In each step, the CRA influx rate was altered to the next value indicated on the abscissa, and a new steady state was calculated. **(A)** The steady-state TNF levels for the acute mode are plotted as ordinate. These were subsequently taken as initial conditions with the exception of the TNF level, which was equated either to 100 or to 0.0001 pM before simulating the system's evolution to steady state in time. The dashed arrow describes the evolution of the TNF level. Taking the final values as initial conditions, the steady-state mode of Copasi was used to confirm that the final condition was stable and steady, with the exception of the states at 1 and 10 fM/min where these initial conditions led to a relaxation to the upper, “chronic” state, as indicated by the small arrows at the *top*. In these cases, initial conditions of 10 pM rather than 100 pM TNF did lead to a relaxation to the lower, “acute” state. **(B)** The same as in **(A)** but now for the chronic branch.

When also computing the control coefficients for the other concentrations, and with one set of exceptions, we mostly (see below) found all control coefficients referring to the dependence of concentrations on process activities to be “moderate,” where “moderate” refers to absolute values not far beyond 2, with 1 being the magnitude of total control of any flux by all reaction activities (57). The exception was for the acute mode of inflammation at one particular magnitude of the influx of antigen, where multiple control coefficients attained high absolute magnitudes. Figure 3A shows that while TNF levels were becoming sensitive to the zero-order reaction rate constant of CRA influx, when that was increased from 0.1 to 10 fM/min, they became ultrasensitive when the influx rate approached the threshold (42) at 16.7 fM/min. Close to this CRA influx rate there appeared to be a singularity where the control coefficients amounted to infinity, corresponding to the vertical jump observed for the acute inflammation branch in Figure 4A.

For CRA influx above this threshold level, where the TNF level had increased to that characteristic of high inflammation (see Figure 4A), the TNF-by-CRA_influx_ control coefficient dropped to zero (Figure 3A). In the high inflammation state, the TNF level was “maximal” at 50 pM and insensitive to the amount of CRA flowing into the system from the outside. In the chronic mode above, at a CRA influx of 0.1 fM/min the network itself sustained a high CRA and FLC, enough to fully saturate and activate the mast cells, leaving no opportunity for CRA influx to affect the inflammation. Figure 3C shows that for the chronic mode of inflammation, i.e., when we stepwise reduced the CRA influx from 30 fM/min down to 0.001 fM/min and computed the steady state at each step, the sensitivity of the TNF level to influx of CRA was indeed zero at first but then regained its sensitivity, but only at CRA influx rates below 0.1 fM/min. This occurred as the high TNF levels of around 50 pM began to decrease (Figure 4B). At CRA influxes below 0.01 fM/min, the control coefficient of TNF levels to the CRA influx rate then approached 2 (Figure 3C), much higher than at the same CRA influx values in the acute mode (Figure 3A). The magnitude of the control coefficients depended strongly on whether the system operated in the acute or in the chronic mode of the system, the TNF level in the chronic mode being much less sensitive to changes in CRA influx at intermediate CRA influx rates and much more sensitive at low CRA influx (Figures 2, 3A,C). This is why we call this a “bi-modal” control analysis.

In the chronic mode, no singularities were observed. This implies that, whereas at CRA influx rates immediately above the singularity in the acute mode at 16.7 fM/min the system jumped from the low inflammation model to the mode of high inflammation, the reverse would not happen as a jump back to the same low inflammation mode at any low CRA influx rate. Once the system was caught in the high inflammation mode, it would stay there. This is the first reason why we called the high inflammation mode the “chronic mode” and the low inflammation mode “acute” (see also below).

Figure 3D shows that, in the chronic mode, TNF levels were proportionally sensitive to the reaction rate constant of TNF production throughout the range of CRA influx rates again, but now for the intermediate CRA influx rates, highly different from what was observed for the acute mode (Figure 3B). Supplementary Table S3 shows all concentration control coefficients for acute inflammation. Control was always distributed over multiple components, and expected summation laws (51) were observed except when variables were close to zero, which caused numerical inaccuracy problems. Supplementary Table S4 does the same for the chronic mode.

### Bistability

The singularities in the control coefficients reminisce a bistable dynamic system (43, 50) in which, for a range of values of a so-called bifurcation parameter, there are two stable steady states. Which of these steady states is attained then depends on the system's initial state, in the simplest case on the initial value of a single bifurcation variable. The dashed arrows in Figures 4A,B show that both modes of steady state were highly stable to TNF perturbations. An increase in TNF to 100 pM, or a decrease to 0.1 fM, was followed by a relaxation back to the steady-state value. Exceptions were the acute states at CRA influx rates in excess of 0.1 fM/min, where these perturbations directed the system to the chronic mode. Still for the acute mode states at 0.5, 1, 5, and 10 fM/min, a more modest perturbation to 10 pM of TNF was followed by a relaxation back to the acute state. The acute mode was locally stable throughout (as was the chronic mode of states).

**Figure 5:**
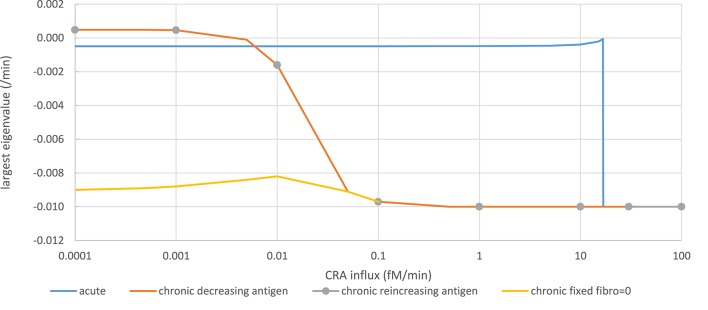
Stability analysis by monitoring steady-state eigenvalues in various network modes. Acute mode (solid blue line): The steady state computed for a CRA influx rate of 0 pM, and a healthy fibroblast level of 1 pM was taken as starting point from which a new steady state was computed after increasing the CRA influx rate to a value on the abscissa successively. The ordinate shows the computed largest (real part of the) eigenvalue. Chronic mode (solid red line): The steady state computed for a CRA influx rate of 30 fM/min, and a healthy fibroblast level of 1 pM was taken as starting point from which a new steady state was computed after reducing the CRA influx rate to the value indicated on the abscissa. The ordinate shows the computed largest (real part of the) eigenvalue. For CRA influxes at and below 0.01 pM, the steady states were reported as unstable, but subsequent time integrations and steady-state computation altered neither the eigenvalues nor the TNF level. Chronic mode re-increasing antigen exposure after maximal reduction (gray dots). From the endpoint of the computations for the red line (i.e., at CRA influx of zero and extremely low TNF and healthy fibroblast levels), the CRA influx was increased; the steady-state eigenvalues were computed and shown as gray circles. Fibroblast-fixed chronic mode (yellow line): Largest eigenvalues for the chronic steady state were computed similarly as for the red line but with the healthy fibroblast level fixed to zero. The computed steady states were now reported as stable.

The model has many more variables however, and physically steady states should be stable toward small perturbations of any variable. An effective way of checking this more complete stability is to calculate the eigenvalues of the system at the steady state. Stability is obtained if the real parts of all the eigenvalues are negative in sign. The stability analysis by Copasi reported that the steady states of the acute mode were stable throughout, i.e., also at the high TNF levels obtained for CRA influx in excess of 16.7 fM/min; their largest eigenvalues were always negative. As shown by the blue line in Figure 5, below the CRA influx threshold of 16.7 fM/min the real part of the largest eigenvalue amounted to −0.0005 /min, corresponding to a relaxation to the steady state within a couple of days. At the more intense inflammation above that antigen-influx threshold, the inflammation was stable at an eigenvalue corresponding to a relaxation in a couple of hours. At the threshold itself the largest eigenvalue became zero, making the slowest relaxation very slow.

In the chronic mode (red line in Figure 5), while reducing the antigen load the eigenvalue remained ~-0.01/min, until at a dosage of 0.1 fM/min it increased to become positive at around 0.0005 fM/min. Accordingly, no stable steady state was reported for CRA influx levels below 0.01 fM/min. This was due to extremely low and variable fibroblast levels. Running the time dependence simulation did not cause the system to move away substantially from these low fibroblast levels and positive eigenvalues, for instance, to the stable steady state corresponding to the acute mode of inflammation. When fixing the healthy fibroblast level in the model to zero, a stable steady state was obtained at the same level of TNF, corresponding to the chronic inflammation mode, the largest eigenvalue decreasing to −0.009/min (yellow line in Figure 5). Moreover, when increasing the antigen dosage from below 0.0001 fM/min back up by successive factors of 10 (gray dots in Figure 5), the eigenvalue retraced its steps along the line corresponding to the chronic inflammation mode as the TNF level itself did (Figure 4B). Notably, the unstable steady state described here for the chronic mode did not correspond to the unstable steady state in between the two stable steady states to be discussed below; it corresponded to one of the two stable steady states. We conclude that in essence (i.e., save a fibroblast level fluctuating at virtually zero), both the acute and the chronic modes of steady state were stable.

### Hysteresis

As already apparent from the above results, bistability can come with hysteresis. Indeed when the CRA influx rate was reduced from 30 fM/min down to rates below 16.8 fM/min, the TNF levels remained high until only at CRA influx levels below 0.1 fM/min did they start to decrease (Figure 4B). For CRA influx rates between 16.8 and 0.001 fM/min, the steady-state TNF level depended on whether the initial state for the computation had been 0 fM/min (the acute mode; Figure 4A) or 30 fM/min (the chronic mode; Figure 4B); the system was hysteretic rather than Markovian, i.e., it remembered its history.

Because in Figure 4B the inflammation remained intensive all the way down the CRA influx rates of 0.03 fM/min, i.e., notwithstanding a 1,000-fold reduction of the antigen challenge, it appeared to be hard to get rid of. Moreover, even though it might seem that the blue lines of Figures 4A,B converged toward a common point at 0.1 aM/min CRA influx and a TNF of 0.5 fM, they did not merge, neither for further reduction of CRA influx rates nor for subsequent re-increase of CRA influx rates; the two lines did not intersect, but crossed, consolidating hysteresis. This further motivates our reference to the line in Figure 4B as the “chronic inflammation” mode. We likewise continue to refer to the steady states depicted in Figure 4A for CRA influx rates below 16.7 fM/min as the “acute inflammation mode.”

Such hysteresis could be explained by the acute and the chronic modes differing from each other in some other property than TNF level at this apparent intersection point. Indeed the fibroblast levels were high (999.5 fM) and very low (reported as 5 10^−37^ fM), respectively, at the crossing point in Figure 4.

We conclude that increasing the antigen load from zero in a naïve system first produces minor “acute” inflammation, which then becomes strong inflammation as the threshold of 16.7 fM/min is transgressed. The system is then in the “chronic” mode. Then, reducing the antigen load to far below that threshold value does not cause a flip back to the “acute” mode even though the corresponding TNF levels can become lower than those in the acute mode (compare Figure 4B to Figure 4A at very low CRA influx rates). The transition from acute mode to chronic mode was *irrevertible*. In the section “Discussion,” we shall discuss how this does not quite correspond to the *irreversible* transition known for simpler dynamic systems (58).

Between antigen loads of 0.5 and 16 fM/min, the stability of the acute mode is already limited. Minor increases in TNF lead to a return to the acute mode, but major ones already induce a flip to the chronic mode of inflammation. This indicates a role of noise in TNF in inducing precocious transitions into the chronic state. Recently, Mavroudis et al. (30) highlighted such a role of noise in the immune response.

### Switching, Irrevertible Transition, and Fibroblast Levels

**Figure 6:**
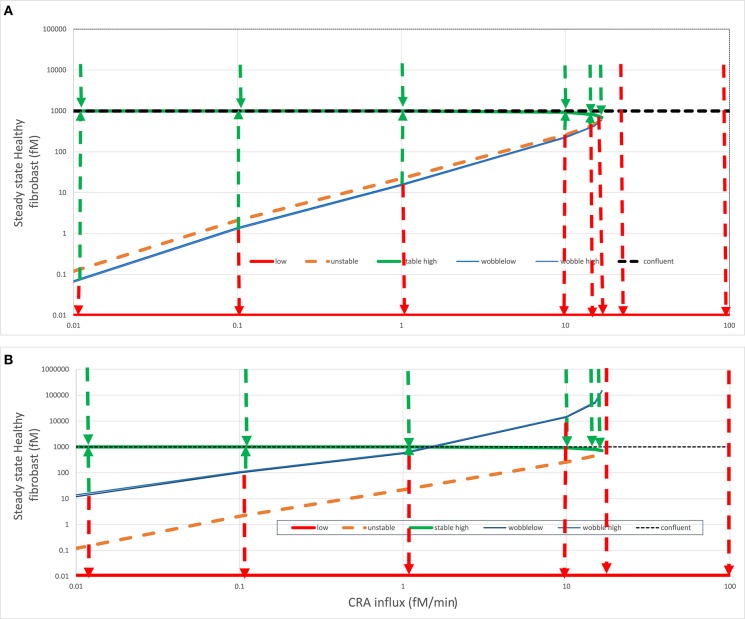
Fates after fibroblast intervention. In **(A)** acute (green line) or **(B)** chronic (red line) steady states (see Figure 4), healthy fibroblast levels were varied and then used as initial states for a calculation by Copasi of the steady-state fibroblast level. This strategy was used to find (1) the healthy fibroblast level corresponding to the unstable steady state (dashed orange line) and (2) the “wobble” fibroblast level (blue line) beyond which the systems transited to the other stable steady state, as also indicated by the dashed arrows. For both **(A)** and **(B)**, the steady state fibroblast level (in fM) is plotted vs. the CRA influx rate (in fM/min). The dotted horizontal line at the fibroblast level of 1,000 fM corresponds to the maximum fibroblast level, which corresponds to complete space filling (3D confluency); all points above that line are biologically (but not mathematically) infeasible. In the chronic steady states, the fibroblast levels were ultralow and, for the purpose of plotting, equated to 0.01 fM. The wobble points depended somewhat on initial states and varied somewhat between calculations, possibly due to the strong temporal dynamics of TNF.

The innate immune system that we here model is complex, and more than one variable is involved in its regulatory loops. In Abudukelimu et al. (42), we already showed how the steady-state fibroblast level also varied in a complex way with antigen load (CRA influx rate). When starting at very low antigen exposures, the naïve system followed the green line in Figure 6A corresponding to the acute mode, with fibroblast levels close to 3-D confluency (1,000 fM). At the CRA influx rate of 16.7 fM/min, this stable mode ceased to exist, and the system dropped to only one of the two stable steady-state modes that remained, i.e., the strongly inflamed one indicated by the red line, with ultralow fibroblast levels, well below the 0.2^.^10^−10^ fM corresponding to a single fibroblast per human body. In a separate run of computations, we therefore fixed the fibroblast levels to zero, producing more stable steady states, but without other significant differences (as also mentioned above).

The green dashed arrows in Figure 6 again show that the acute-mode states were stable against substantial perturbations, now of fibroblast levels. Below the CRA influx threshold, perturbation of the fibroblast level to 10,000 was followed by relaxation to the steady-state level just below 1,000 fM as did perturbations to the 3D-confluency level of 1,000 fM (not shown but implied). Reducing the fibroblast levels to some extent was also followed by a relaxation back to the acute-mode steady state, but the extent of the perturbation for which this was true diminished as the CRA influx approached the threshold rate (of 16.7 fM/min). The perturbed fibroblast level above which the relaxation proceeded back to the acute mode is indicated by the blue “wobble” line in Figure 6A. For a stronger perturbation of the fibroblast level, the system switched to the chronic mode, as indicated by the red dashed arrows pointing down to the corresponding red “chronic” line. Above the threshold CRA influx, all such relaxation was to the chronic mode with ultralow fibroblast levels.

In simple bistable systems, there is an unstable steady state “in between” the two stable states, i.e., for a given value of the bifurcation parameter, there is a value of the bifurcation variable that corresponds to a state where the time derivatives of all variables are again zero, but at least one eigenvalue is positive. By using various starting values for the fibroblast level, we found such unstable steady states through Copasi and have indicated these by the dashed orange line in Figure 6. In simple bistable systems, one can move between stable steady states by changing the bifurcation variable just a bit across its unstable steady state value. The figure suggests, and more detailed computations (not shown) confirmed, that this was not the case here: the blue “wobble” line is always below the orange unstable states line in Figure 6A. Starting from an acute inflammation state, we had to reduce the fibroblast level significantly below its unstable steady state value in order to provoke “switching,” i.e., relaxation to the chronic rather than the acute mode. Just reaching the unstable steady-state value was not enough; the system was conservative in that it seemed to resist the flipping between stable steady states.

When starting from the acute mode (Figure 6A), the distance between the unstable steady states and the wobble points was small, but when starting from the chronic mode (Figure 6B) this distance was much larger, close to a factor of 100. Accepting that realistically the fibroblast level cannot be brought to concentrations in excess of 1,000 fM, chronic inflammation at CRA influx rates between just above 1.5 fM/min (i.e., less than one tenth of the threshold flux) and that threshold flux (16.7 fM/min) cannot be switched back at all to the acute mode by maximum repopulation with fibroblast. Since 1.5 fM/min might be the minimum antigen load that can be achieved in most cases, this could imply that, although the acute to chronic transition is not irreversible in theory, it cannot in practice be reversed, yet another reason to call the transition “irrevertible.” Above we showed that it cannot be reversed by reduction of antigen influx alone, and here we show that it cannot be reversed by fibroblast implantation alone.

This irrevertibility is a consequence of the complex non-linear nature of the innate immunity model. The same complexity causes more paradoxical phenomena. In the CRA influx range between 1.5 and 16.7 fM/min, perturbation of the fibroblasts from the chronic level to a level above the wobble line is followed by a relaxation back to the acute mode. Paradoxically, this relaxation passes the fibroblast–relaxation path subsequent to a perturbation to slightly below the wobble level, which relaxes to the chronic state (see the red and green dashed arrows at 10 fM/min in Figure 6B).

The origin of these paradoxical behaviors resides in the complexity of the system. The fibroblast level is only one of the bifurcation variables. When bringing the fibroblast level just across the magnitude of the unstable steady state, that level itself may indeed be attracted to the other steady state, but the other dynamic variables, such as TNF and FLC, are still at magnitudes that favor relaxation to the original steady state. We have tested this insight by simultaneously moving two bifurcation variables in the direction of the transition to the other steady state, in this case, from the chronic to the acute mode. Table 1 shows that, whereas changing only TNF or only the fibroblast level in this computation did not cure the chronic inflammation *in silico*, their combination did. That this corresponds to cooperativity is shown by the result (Table 1) that much stronger treatment in terms of modulating either factor alone did not cause the transition from chronic to acute mode.

**Table 1:** Computed effect on chronic inflammation of treatment with fibroblast implantation and/or TNF reduction.

**Treatment**		**Result**		**Cure?**
**TNF treatment pM**	**Fibroblast implantation fM**	**Fibroblast fM**	**TNF pM**	
None	None	2.10^−39^	49.5	
None	0–200	1.10^−100^	49.5	No
49.9–10	None	6.10^−245^	49.5	No
49.9–10	0–200	998	0.02	Yes
None	0–400	9.10^−7^	49.5	No
49.9–0	None	9.10^−100^	49.5	No

*The low fibroblast levels reported by the program essentially equal zero. CRA influx rate was 1 fM/min throughout*.

### Jumping the Bistability Barrier: Fibroblast Proliferation

**Figure 7:**
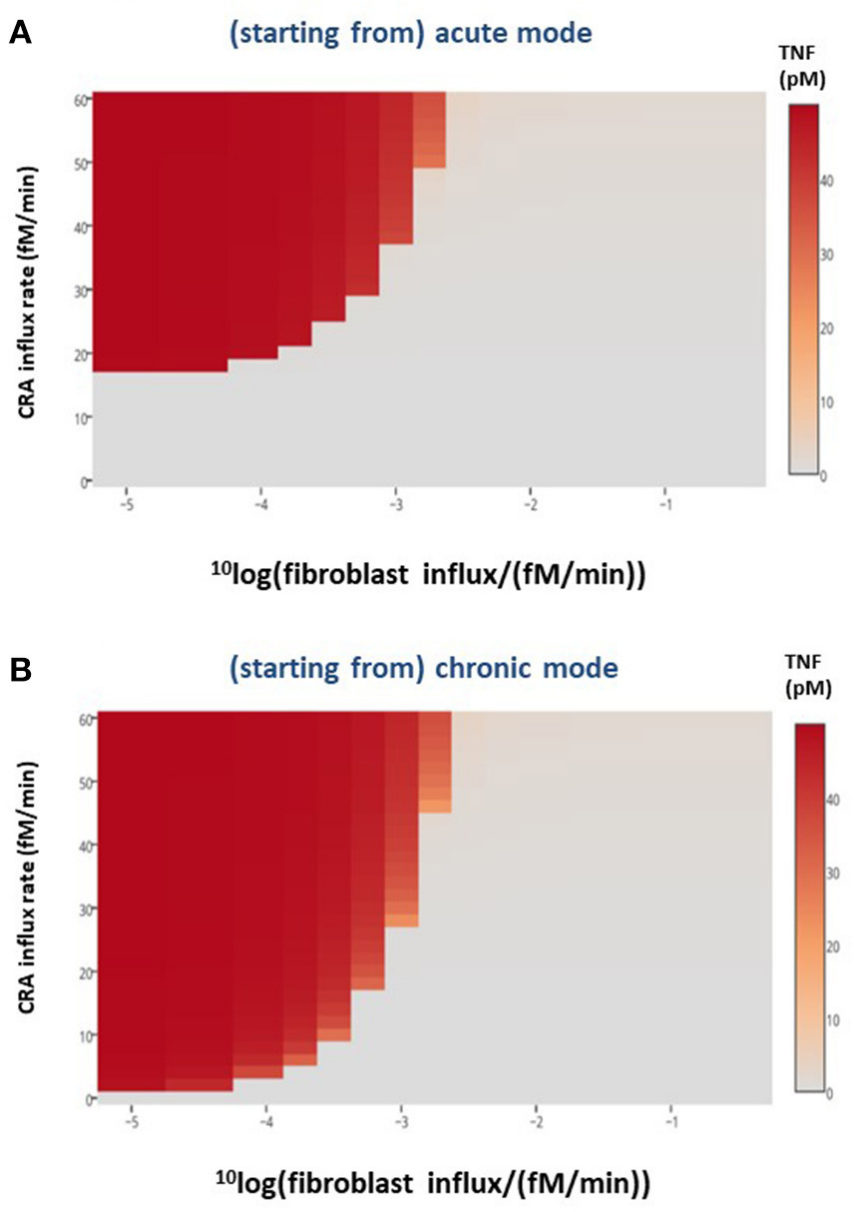
Healthy fibroblast influx and inflammation, *in silico*. For various CRA influx rates and various influx rates of healthy fibroblasts, the stable steady-state TNF level was calculated for the **(A)** acute and **(B)** chronic inflammation modes, respectively. The TNF level as a function of CRA and fibroblast influx rates are presented as a heat map, the color reflecting the corresponding steady-state level of TNF (in pM), as indicated by the bar on the right-hand side.

In the preceding section, we showed that merely adding a batch of fibroblasts to our chronically inflamed model rarely switched it back to its acute mode. This was presumably because the added fibroblasts were subject to the toxicity of the strongly elevated TNF and disappeared rapidly. This suggested that a sustained influx of fibroblasts into the inflamed area might be more successful in reverting the chronic inflammation. The heat map in Figure 7B shows that indeed allowing fibroblasts to grow into the system at a fixed low rate already affected chronic inflammation at low CRA influx levels, but not yet much at high CRA influx levels. Fast fibroblasts ingrowth (i.e., above 0.3 10^−2^ fM/min) completely suppressed chronic inflammation at all CRA influx rates (Figure 7B). Figure 7A shows that the low fibroblast influx rate (10^−5^ fM/min) had no effect on acute inflammation, whereas a thousand-fold faster fibroblast ingrowth suppressed high inflammation in the acute inflammation mode altogether, preventing the upsweep to high TNF levels at CRA influx above 16.7 fM/min (compare Figure 7A with Figure 7B).

### Jumping the Bistability Barrier: Targeting IgLC or IgE

Another component of the positive feedback loops in the innate immunity model is the immunoglobulin light chain (IgLC), as freely circulating light chains or, in a different incarnation of the model, as IgE. We had already found *in silico* that an IgLC-neutralizing peptide reverted chronic inflammation to its acute counterpart in the sense of reducing the TNF level (42). It did this only temporarily however; after some time, TNF increased again, ultimately returning to the TNF level characteristic of chronic inflammation. The results of the preceding section suggest an explanation: the system might be trapped irrevertibly in the stable chronic inflammation state. Accordingly, the anti-IgLC peptide might then temporarily reduce the inflammation, but it might be impossible for the system to escape from the attractor of the chronic state in this way.

**Figure 8:**
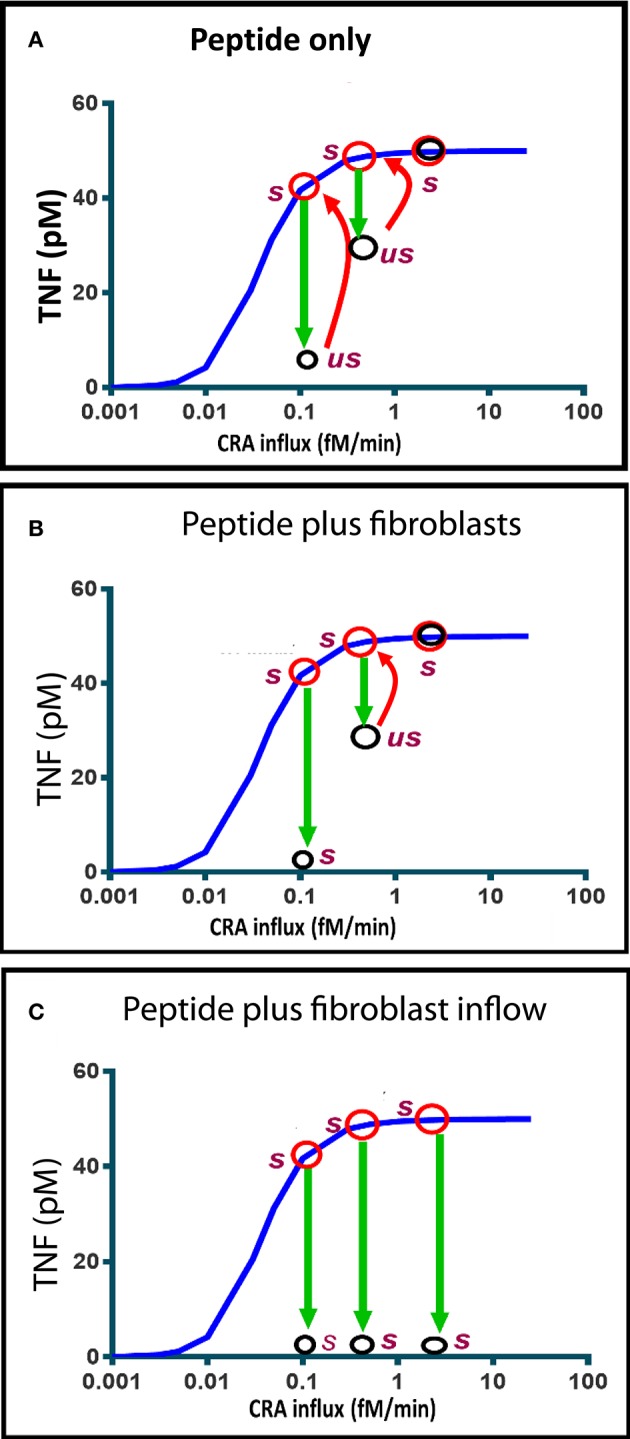
Computed time dependence of chronic inflammation subjected to anti-IgLC peptide therapy with or without fibroblast supplementation. The steady-state chronic inflammation level of TNF (red circles) was computed for CRA influxes of 0.1, 0.5, and 3.0 fM/min, respectively, each time starting from the initial state at 30 fM/min. **(A, B, C)** Anti-IgLC peptide was then added to a concentration of 1 nM. In **(B)**, fibroblasts were added as well to a concentration of 5 fM. In **(C)**, fibroblast influx was started instead at a rate of 0.01 fM/min. The open black circles indicate the TNF levels attained after 6 h. *us* refers to “unstable” and *s* to stable. Further development (if any) in time of the TNF during the subsequent day is indicated by the red arrows. The blue line represents the chronic inflammation steady states.

At three different CRA influx rates, we checked whether indeed the state produced by addition of the peptide (which caused the decrease in TNF level shown by the green downward arrow in Figure 8A) was unstable. We found that for the two cases where the peptide had lowered the TNF level, the situation was unstable 6 h after peptide addition; where CRA influx was as high as 3 fM/min, the TNF level was as high as in the beginning. The red arrows show that, for all three cases, by 1 day after peptide addition the simulated TNF level had returned to its initial high-level characteristic of strong inflammation.

Figure 8B shows that if fibroblasts were added as well, the state achieved 6 h after peptide addition was stable and characteristic of reversal of the chronic inflammation to the acute mode. The absence of a red arrow means that there was no subsequent change. This all happened at a CRA influx rate of 0.1 fM/min. At a ten-fold higher CRA influx, the peptide addition only produced a transient unstable state, which reverted to full inflammation by 1 day after peptide and fibroblast addition. When instead of a single fibroblast addition fibroblasts were infused continuously, the chronic inflammation was reversed at all three CRA influx rates of the computations (Figure 8C). We conclude that influx of fibroblasts stabilizes the low inflammation state induced by the peptide drug.

## Discussion

Our dynamic model of the innate immune system in inflammation (42) exhibited various features that were at odds with the usual “watchmaker” models of metabolic pathways (59–62). It exhibited a preference for one of two families (modes) of states. In the present paper, we report some more peculiar observations that the model makes and that may well speak to innate immunity itself, such as recurrent bursts of inflammation, hysteretic behavior, the role of an apparent bystander (fibroblasts) in reverting the preference for the “chronic” state, and the potential of such a bystander for making peptide therapies definitive rather than unreliable.

An example of how stability analysis can help understand immune-system-related pathologies is the work by Murase et al. (63), who reported that pathogens can destabilize the interior balance of a model that represented pathogen–immune interaction dynamics (63). In pharmacokinetics, steady-state drug effect models have been used to estimate the robustness of drug dose–response relationships and considered essential to phase 2 drug development (64). Such a procedure should also be relevant for the future applications of peptides interfering with immunoglobulin light chains (40, 42, 65). These and other examples made us employ stability and control analysis to examine what determines the preference for the chronic inflammation state, how strong this preference is, what the preference implies with respect to the dynamic and hysteretic properties of inflammation-associated diseases, whether the preference and its implications are inescapable, and how anti-IgLC peptide therapy might be improved.

### Limitations and Ramifications

We carried out these analyses in a mathematical model of the innate immune system rather than in an experimental model system. We did this because the mathematical model gives complete control and allows unlimited invasive experimentation *in silico* to test ideas and concepts, whereas *in vitro* models are much harder to manipulate and analyze quantitatively and robustly. The mathematical model that we used has been constructed by our bottom-up methodology (42). We translated the individual processes of the known network into reaction equations and rate equations. We then integrated these equations in time, solved them for steady-state concentrations and fluxes, and performed stability and control analysis using the biochemical pathway simulator Copasi to obtain numerical solutions (52). We confirmed the stability or lack of stability of steady states reported by Copasi's “stability analysis” option by computation of the time dependence upon perturbations away from the steady state, by analysis of the eigenvalues of the Jacobian, by performing control analysis, by changing the settings of the Copasi software such as step size, and by changing the initial values of free variables. The results suggested a robust and regular behavior of our numerical methodology, with three possible exceptions. Firstly, in the acute mode at a CRA influx around 16.7 fM/min, control coefficients tended to infinity (Figures 3A,B). This was caused by a bifurcation turning the system from a bistable to a mono-stable system and not by a numerical insufficiency. The low inflammation steady state jumped up to its high inflammation steady state for an infinitesimal increase of the CRA influx rate (see Figure 4). Secondly, the fibroblast levels reported for the chronic mode were extremely low in absolute magnitude and sometimes negative in value. We showed however that if the fibroblast levels were set to zero (and left fixed or variable), the model's behavior remained unchanged, the positive eigenvalues (which were associated with the fibroblast levels) disappeared, and Copasi reported the same control coefficients. Moreover, the summation laws of metabolic control analysis (49) were met (see Supplementary Tables S3, S4). We conclude that the phenomenon is due to the autocatalytic nature of fibroblast growth and the inability of digital computers to end up at true zero values. The third special observation was that the stepwise decrease of CRA influx with at each step the steady state being computed, when followed by a stepwise increase of CRA influx, led to a retreading of the steady states' trajectory of the CRA downward chronic mode rather than a progression along the acute mode. We showed that this was not due to a numerical anomaly either but rather to the fact that although the TNF levels of the chronic and the acute mode crossed each other, the fibroblast levels were highly different. We conclude that our results are not compromised by numerical limitations.

The absence of numerical ramifications does not mean that our results are relevant for innate immunity in practice. Due to the very complexity of the total innate immune system, we have had to keep our model simpler than reality. Limitations include the inability of mast cells and B cells to proliferate, the lack of compartmentation, the absence of many cytokines, the simplifications in where the relevant antigens come from, and the absence of adaptive immunity. In addition, the kinetic equations and parameter values that we used were simple and, though founded in realistic assumptions (42), were limited in experimental foundation of their magnitudes.

Our earlier paper on the same model of innate inflammation (42) contains a rationalization of the model species, model equations, and parameter choices made. A model of this complexity cannot at present be fully validated experimentally. Such a validation would require hundreds of experimental manipulations with highly accurate functional measurements in some 10 cellular compartments that are ill-accessible experimentally. Therefore, we have to be satisfied for the moment with validation in the sense that experimentally observed emergent behavior is predicted successfully by the model (10). After all, this is the status of perhaps the best validated complex models we know: ability to simulate the (lack of) emergent behavior of mutants (66). More complete experimental validation is only possible for much simpler metabolic models such as our *E. coli* ammonia assimilation model, and even then we needed special methods to deal with uncertainties (67).

The behavior of our model is however similar to innate immunity in practice, which is what one may consider a mild validation: it is able to deal with microbial infections; it exhibits acute and chronic modes with persistence of the latter; it affects TNF strongly; and it shows that anti-FLC peptides should be expected to have effects but that these effects are conditional (65). The model describes fibroblast levels of up to 1 nM, which corresponds to a realistic cell volume of 1.7 pl and contact inhibition. Its mast cell and B cell concentrations are 10,000 and 1,000 times lower, respectively. At maximum inflammation, the TNF levels amounted to 50 pM. This corresponds well with the observation that TNF activates cells at picomolar concentrations, with its receptors having one K_D_ around 20 nM and a second K_D_ of 400 pM (68). The TNF levels that we predict in the high inflammation mode of 50 pM should not yet saturate these receptors. Serum TNF levels of healthy individuals are around 0.4 pM, with levels around 1 pM for people with inflammatory conditions (69), which we imagine to stem from 50-fold dilution out of inflamed areas into the blood stream. The other cytokines, MMP7 and MMP8, occur at grossly similar levels in our model results, in agreement with reality in serum (69). Of course these correspondences between model predictions and reality do not prove the model to be correct; a wrong model can lead to right predictions. In conclusion, the model is validated in this and our previous paper to some extent, but incompletely, by the correspondence of its behavior to reality. Because its conclusions are of interest, the model should be further validated and perhaps improved in a more comprehensive future study also engaging in new experimentation. Ultimately, one would like to achieve a version of this model that is in close correspondence with all experimental data in innate immunity, as well as with the other existing mathematical models.

Because we were not estimating (fitting) model parameters in our paper, identifiability was not an issue. Our model predictions are based on the most reasonable parameter values (see the rationalization in our previous paper) fixed at the onset and not adjusted in order to obtain a better fit. Our control analysis shows moderate control coefficients, suggesting limited parameter identifiability. Multiple parameter values lead to the same qualitative conclusions. This has the disadvantage that the model parameters are not identifiable, but the advantage that our conclusions about the *in silico* model should have a higher probability of being relevant for the situation *in vivo*.

Moreover, the model appears to be useful; it makes predictions of how modifications of therapies might improve their impact, with suggestions for applications such as in allergy treatment. It should help that not every detail is essential for our observations and conclusions. Important are the positive and negative feedback loops in the innate immune system and the fact that, for any increase in the number of fibroblasts, fibroblasts need to be present. Adding more features of the actual innate immune system will add more features of this type as well so that we expect that the (in)stability aspects we modeled here will persist. This implies that interference with the innate immune system with medicinal drugs, such as the IgLC-binding peptide modeled here, should be effective equally conditionally when other targets are chosen, such as TNF (an effective target in the treatment of rheumatoid arthritis (49, 70) or one of the MMPs.

### Complex Stability of a Model of Innate Immunity: State Trapping

A most pertinent model observation was that treatment of the high inflammation state with anti-IgLC (anti-IgE) peptide initially appeared to be effective in inducing a switch to the low inflammation state, but the therapy became ineffective after a much longer time. The present paper has clarified this. Firstly, we verified that the model is home to two modes of stable steady states, which overlap in terms of corresponding parameter values, i.e., bistability was occurring. Secondly, we found that the model is somewhat out of the ordinary; redressing the CRA influx from the high magnitude characteristic of the strong (“chronic”) inflammation mode all the way back to zero did not cause the system to jump back to its acute mode.

This transition from acute to chronic mode is what we call an irrevertible transition. The transition is “irreversible” neither in the sense of non-equilibrium thermodynamics (57) nor in the sense of simpler bistable systems (49) with a single bifurcation parameter requiring unrealistic negative values for the reverse transition. We established that the TNF levels of the acute and the chronic branch neither merged at low values of CRA influx nor stayed away from each other as they do in the simple case of bistability with a simple irreversible transition (49). They crossed each other (compare Figure 4A to Figure 4B around CRA influx rates of 0.0001 fM/min).

Looking at how, for the chronic mode of steady states, the fibroblast level varied with CRA influx rate, we found that the fibroblast level was always very close to zero and did not recuperate. Even though the peptide drug decreased the TNF level to a value close to that characteristic of the low inflammation state, it did not increase the fibroblast level from the very-close-to-zero level characteristic of the highly inflamed state back to the high level characteristic of the low inflammation “acute” mode of states; thereby, the peptide treatment did not completely undo the irrevertible transition to the high inflammation state. This suggested that increasing the concentration of fibroblasts might be able to revert inflammation from its high chronic mode to its low acute mode, and indeed *in silico* addition of fibroblasts or a constant influx of fibroblasts was able to achieve this.

The complexity of the irrevertible transition was greater than this however. The actual transition point from acute to chronic and much more so that from chronic to acute did not correspond to the magnitude of the fibroblast concentration at the unstable state. We called the transition points “wobble points” (see the blue lines in Figure 6). The reason for the transitions from “chronic” back to “acute” or *vice versa* being determined neither by the TNF nor by the fibroblast unstable steady-state value alone is that the bifurcation is determined by multiple state variables rather than one. We witnessed a complexity in our innate immunity model that derives from its multidimensionality, with multiple molecular and cell species determining fate. Indeed altering both the TNF and the fibroblast levels at the same time, we more readily switched the system (Table 1).

The chronic inflammation branch of states was particularly robust to ordinary fluctuations and perturbations, thereby requiring very special perturbations for *in silico* therapies to be successful. Figure 6B reveals another limitation to the revertibility of the chronic mode: for CRA influx rates between 2 and 17 fM/min, the wobble point exceeded 1,000 fM, i.e., the maximum fibroblast concentration corresponded to filling all space with fibroblasts. This makes the chronic state indeed an irrevertible trap for the innate immune system.

### Implications for Novel Therapies

To the extent that our model corresponds to reality, this conclusion could have important implications for the management of innate immunity and for potential therapies of its diseases. Anti-IgLC peptide therapy may work temporarily but not definitively, while sufficiently intensive fibroblast therapy should cure definitively, but only if the cross-reactive antigen load is smaller than what it might be. Strategic combination therapies should prove more successful, and planning these with the dynamic models of the type that we developed here might be of help. Most probably, a triple therapy should be advised, i.e., a reduction in the antigen load, some agent interfering with a cytokine or IgLC, and a possibly sustained enhancement of fibroblast density.

The control analysis we performed for the chronic state confirms this difficulty for therapies of chronic inflammation. Processes other than TNF synthesis and TNF washout exercised no control on the TNF level in the chronic steady state, confirming that there was no target for which limited interference could affect inflammation. If anti-TNF is to work definitively, very high doses should be required. This was different for the acute mode of inflammation, where a great many processes exercised such control, which suggests that by focusing on any of the many processes with the high control coefficients one should be able to design many medicinal drugs preventing the inflammation from becoming chronic. Also here prevention should be much better than therapy.

Our prediction that ultimately anti-IgLC therapy alone may not work may not quite be realistic. In actual cases, there will always be ingrowth of fibroblasts, and therefore one would obtain in any practice the situation of a combined therapy with fibroblast ingrowth and anti-IgLC peptide. Our conclusions should still be taken to mean then that activation of fibroblast ingrowth (or in practice activation of corresponding stem cells) should be considered a strong candidate adjuvant therapy. Another ramification is that with time after the first challenge, the adaptive immune system will become active and take over much of the role of the innate immune system. Therefore, the flares of microbial growth and the switch to chronic inflammation may be moderated by the adaptive immune system taking away the antigenic stimulation that lies at their bases.

### A Special Type of Bistability

There are many models of bistable dynamic systems. The present model of innate immunity is however special. The transition to the chronic state is irrevertible in the sense that (1) the chronic mode does not revert to the acute mode, not even at the point where both the bifurcation parameter CRA influx and the bifurcation variable TNF are the same for the acute and the chronic mode, (2) this is not an irreversible transition due to the fact that the bifurcation parameter cannot become negative, (3) TNF reduction cannot definitively revert the acute to chronic transition, (4) often fibroblast addition cannot do this either because the concentration of fibroblasts cannot exceed its physical limit, and (5) dual intervention can revert the irrevertible transition, but only in special ways, sometimes requiring a third intervention (e.g., fibroblasts, anti-IgLC peptide, antigen level).

### Robustness of Innate Immunity

We found that the type, more than the magnitude, of the inflammatory response depended on the influx of CRA protein. Below a certain threshold level, the cross-reactive antigen did not elicit much of an inflammatory response, i.e., the steady-state TNF and fibroblast levels were low and high, respectively, and hardly affected by the CRA influx rate. Above a certain threshold level, varying the CRA influx did not affect much the steady-state magnitudes of these two variables either, but now they were low and high, respectively. At the transition, there was a very sudden increase in TNF level and an equally sudden drop in fibroblast level. The control analysis for the acute branch confirmed this. The TNF and fibroblast levels were fairly insensitive to the influx of cross-reactive antigen throughout but became ultrasensitive at and around the transition point. The control coefficients were quite different for the chronic branch. There they were moderate throughout; there was no ultra-sensitivity anywhere. Our heat map of the dependence of the TNF levels on both the CRA influx rate and the fibroblast influx rate confirmed the step transition, now as a function of both the CRA influx rate and the fibroblast influx rate. We conclude that the chronic inflammation branch of the model is highly robust all over. The acute branch is also robust, except near and beyond the critical CRA influx where it transits to the chronic inflammation branch. We are here finding the emergence of phenotypic stability in the face of perturbations of physiological states, with two alternative such states, i.e., acute and chronic inflammation, phenotypic bistability therefore.

An exception was found for the case where the model's innate immunity was activated by a bacterial infection. There we found a virtually stable state after the immunity had dealt with the infection, but every now and then bacteremia seemed to recur. When we tried to cure this (as always, *in silico*) with a sustained influx of antibacterial protease, we found that this seemed to aggravate the situation. The frequency of the spurious emergence of bacteria increased rather than decreased, although indeed the amplitude decreased. Only at high protease influx rates did the pulses of bacterial growth vanish. The model of innate immunity was only marginally stable except when there was excess protease. This may be of interest when trying to understand the re-emergence of bacterial pathogens after apparent silence, such as in the case of tuberculosis. These cases of re-emergence occurred without drug resistance arising and without antigenic variation (71), two effects which in reality will add to the effect modeled here.

### An Important Role for Bystander Cells

The stroma of tissues contains fibroblasts. These cells might seem to serve household functions only; they manufacture protein fibers and synthesize extracellular matrix and collagen. They also fill up the space of the tissue, and when there is empty such space, they grow until they become contact-inhibited again, ensuring filling of the tissue space. In this process, the fibroblasts secrete and respond to growth factors. We made this tissue regeneration process operational *in silico* by including the simulation of the regrowth of healthy fibroblasts. The other side of the same coin, i.e., the innate immune system's response to the growth and influx of healthy fibroblasts, is a novel topic, which we also tried to deal with here. In our simulations, a paradox then emerged. Rather than being a background/household factor, healthy fibroblasts seemed to be essential for switching the system from a disease state to a healthy state. Without their activity, anti-IgLC, which was considered a therapy *in silico*, did not produce a permanent effect. For the effect of anti-IgLC to be permanent, healthy fibroblasts had to be fluxed into the system or a sufficiently high single dose of these cells had to be given. Because our model was also simulating the lymph system and its tendency to cleanse tissue, the anti-IgLC peptide was only present temporarily in the system, whereas even a single dose of fibroblasts generated progeny and thereby provided for a continuous influx of more fibroblasts. The resolution of the paradox that we mentioned above is complex but may reside in the fact that healthy fibroblasts secrete both anti-inflammatory (such as MMP8) and pro-inflammatory (such as MMP7) factors, whereas dying fibroblasts secrete more pro-inflammatory factors (72, 73). Inflammation causes the number of dying fibroblasts to increase, which then puts in motion a positive feedback loop. More research should put the details of this explanation in place or replace the explanation with a better alternative.

Herewith healthy fibroblasts are predicted to have a therapeutic effect. Healthy fibroblasts alone should sometimes be able to cure disease, but only when the immune disease is chronic. In our model, ingrowth of fibroblasts had little effect when the system was in the acute inflammation branch. The acute mode of inflammation is already saturated with healthy fibroblasts, and this is why the curves with and without fibroblast ingrowth showed overlapping TNF activity (Figure 7A for CRA influx rates below 17 fM/min). However, when TNF was there as output response of the chronic inflammation, the TNF curves with and without fibroblast ingrowth showed a different outcome at most CRA influx rates (Figure 7B).

### This Model's Novelty

There are quite a few mathematical models of immunity, but fewer such models of innate immunity. Other than the one we developed and used here, there are no mathematical models that address the (lack of) ability of a peptide drug interfering with IgLC (or IgE) to revert chronic inflammation definitively. This non-robust ability has been the subject of a set of experimental studies by one of the authors of the present paper [(38, 39, 65); see also (41)] and served as the inspiration and guidance for both the present and the previous paper (42). The present paper comes with another novelty however; it provides for an explanation of the faltering effects of this type of peptide as well as a number of suggestions on how to improve the robustness of this type of therapy. Other than just in the bistable nature of innate immunity that has also been discussed by other models although not for this peptide, the novel explanation resides in the special irreversibility of the transition from acute to chronic. Novel is also the demonstration that a dual or even triple intervention should be needed to revert the system from the chronic to the acute state, i.e., reduction in antigen load, the peptide interference with the IgLC, and the implantation of fibroblasts. The consideration of the influence of fibroblast levels, in relationship with their levels at full cell confluency, is also new. Cockrell and An (74) recently also reached the conclusion that multi-dented therapies are needed to deal with sepsis. They built a reinforcement learning workflow for designing corresponding therapies (29), but they do not analyze the source of the complexity in the special type of irreversibility nor do they address the use of anti-FLC peptides.

Some of the other models present *in silico* clinical trials in phenomenological (26, 75) or more mechanistic target (27) terms and address anti-TNF agents, yet other models address the role of mimicry in autoimmunity (76). Pigozzo reviewed the various types of models in existence and then developed their own (22). The model developed by Smith dealt with pneumococcal infections of the lung but did not analyze complex stability properties nor did it report flares of resurgent inflammation (77). Sontag developed an immunology model that exhibited complex stability properties, but this model was phenomenological, i.e., formulated in terms of a few abstract properties rather than being mechanistic as the model used here (37). Its main application was the interesting non-unique relationship between immunological activity and tumorigenesis. Eftimie and Hamam modeled the non-robustness of tumor cell elimination by macrophages due to the presence of two types of CD4+ T cells (35). They performed a sensitivity analysis and observed highly complex bistability, but not of the type discovered here, and in a four-variable model. In a well-documented paper, Qomlaqi et al. (36) modeled adaptive immunity in immunotherapy and showed how efficacy depended on dosage dynamics, but without analyzing the complex stability of the system. Kumar developed a three-component model that simulated the dynamic response of the immune system to a pathogen (54). Their model predicted oscillations of the pathogen level, which were similar to the “flares” of recurrent inflammation observed by our simulations, without reporting however the paradoxical increase in frequency with protease treatment. Their model was small enough to perform an analytical analysis of its eigenvalues. This was an advantage for developing a higher-level concept, but failed for us, where we needed more detail which we could handle by numerical analyses using Copasi. Reynolds et al. did an extensive bifurcation analysis of a model with four somewhat abstract variables (26). They also found bistability and a jump from low to high inflammation for increasing antigen load (represented by the growth rate of the pathogen) and a different trajectory when the antigen load was decreased subsequently. Activation of their anti-inflammation variable also had a time-dependent effect. Some of the same authors also published a more detailed and mechanistic model successfully predicting transient changes in cytokines upon the onset of inflammation (78). Neither paper addressed the same complexity of the irrevertible transition to the chronic state nor the difficulties of reverting back to the acute mode of inflammation such as through anti-IgLC peptide. Mavroudis et al. (30) recently developed a phenomenological model of immunity in which tissue damage played a role, as in our model of 1 year before them (42). They however also highlighted the role of noise or cell–cell diversity (79), which we do not do here, although Figure 4A suggests that noise could be important.

Our elucidation of the role of a type of bistability that has a complex irrevertibility, the introduction of wobble points in addition to unstable steady state, the shift away from an anti-TNF agent to an anti-IgE/anti-IgLC peptide, the explicitness in terms of molecules and mechanisms, the control analysis and concomitant drug target identification, and the role of fibroblasts as much-involved bystander cells are new as compared to the existing mathematical models.

## Conclusion

The dynamic model of innate immunity analyzed here can help to understand various complex phenomena related to inflammation and suggest new multi-dented therapies. Further tuning of the model by interacting with new experimental data and uncertainty quantification (80) should show whether the understanding suggested in this paper is definitive.

## Data Availability Statement

The datasets generated for this study are included in the Supplementary Material and are available on request to the corresponding author.

## Author Contributions

AA developed the model and carried out the simulations together with HW, and wrote the paper together with MB and HW. MB checked the model, assisted with writing the paper, and supervised the work together with HW. FR advised on the immunology with respect to the peptide drug. NS contributed to the design of the network. RS checked the models and computations and the text of the supplemental material written by HW. HW developed the model and performed simulations together with AA, wrote the paper together with AA and MB, supervised the work.

### Conflict of Interest

The authors declare that the research was conducted in the absence of any commercial or financial relationships that could be construed as a potential conflict of interest.

## Supplementary Material

Data Sheet 1 Models and they way in which they lead to the figures of this paper are presented in this file, in conjunction with Data Sheet 2.

Data Sheet 2 The Copasi and SBML files, as well as the Copasi software used. Live model version is available in JWS-Online through https://jjj.bio.vu.nl/models/?id=abudukelimu.

Data Sheet 3 Supplementary Table S3.

Data Sheet 4 Supplementary Table S4.
