# Supplementary material for: Complex Stability and an Irrevertible Transition Reverted by Peptide and Fibroblasts in a Dynamic Model of Innate Immunity
Source: Front Immunol. 2020 Feb 14;10:3091. doi: 10.3389/fimmu.2019.03091 (PMC7033641; doi:10.3389/fimmu.2019.03091)
Supplement: Data Sheet 1 — Models and they way in which they lead to the figures of this paper are presented in this file, in conjunction with Data Sheet 2. [file Data_Sheet_1.pdf]

Supplementary material to:

Complex Stability and an Irrevertible Transition reverted by Peptide and Fibroblasts in a dynamic model of Innate Immunity

Abulikemu Abudukelimu[1,2], Matteo Barberis[1,3,4], Frank Redegeld[5], Nilgun Sahin[2], Raju P. Sharma[2], and Hans V. Westerhoff [1,2,6.7]*

[1]Synthetic Systems Biology and Nuclear Organization, Swammerdam Institute for Life Sciences, University of Amsterdam, Amsterdam, The Netherlands

[2]Molecular Cell Physiology, VU University Amsterdam, Amsterdam, The Netherlands

[3]Systems Biology, School of Biosciences and Medicine, Faculty of Health and Medical Sciences, University of Surrey, Guildford, Surrey, United Kingdom

[4]Centre for Mathematical and Computational Biology, CMCB, University of Surrey, Guildford, Surrey, United Kingdom

[5]Division of Pharmacology, Department of Pharmaceutical Sciences, Faculty of Science, Utrecht University, The Netherlands

[6]School for Chemical Engineering and Analytical Science, University of Manchester, United Kingdom

[7]Systems Biology Amsterdam, VU University Amsterdam, Amsterdam, The Netherlands

***Correspondence: Hans V. Westerhoff, H.V.Westerhoff@UvA.NL or Wff@SysBA.NL**

Table of Contents

Folder with extra information 3

The standard models 3

TNF and MMP7 concentration upgrade of the models 4

Sensitivity analysis and control analysis 5

(Re-)computation of Figures and Tables 5

Numerical aspects 9

Table S1. Specification of the standard model, set to compute innate inflammation at zero CRA influx rate. 10

Table S2. Concentration of various species at steady state and at 4 days into the time course simulation. 13

Table S3. Sensitivity coefficients for the acute inflammation mode (included as separate supplementary file: datasheet 3) 14

Table S4. Nonzero/non-infinite control coefficients of the chronic inflammation mode (included as separate supplementary file: datasheet 4) 15

Folder with extra information

Background information concerning the various Figures and Tables of this paper is provided in a folder with a name beginning with 'Background', which includes the Copasi files enabling to compute them, also in SBML form, as well as the figures in original form.

The standard models

COPASI 4.23 (Build 184, for Windows 64 bit) was (mostly) used and is included in the Background folder. The standard model for the acute mode of inflammation is encoded by the Copasi file 'CRA 0 initial SS acute inflammation.cps'. This model is described in Table S1. It is also available in SBML form through https://fairdomhub.org/models/639 . Simulating through JWS online, one should set metabolite 18 to 1 to circumvent what appears to be a bug. The Copasi form is stored as https://fairdomhub.org/models/638 . Before this paper was accepted for publication these models were visible, not downloadable, but the 2018 model can be used: https://fairdomhub.org/models/640, which has the doi http://doi.org/10.15490/FAIRDOMHUB.1.MODEL.640.1 A calibrated version of the chronic inflammation model for the present paper (in the sense that the TNF and MMP7 concentrations are realistic [see below], has been uploaded to the same hub and has doi: http://doi.org/10.15490/FAIRDOMHU B.1.MODEL.645.1 and for the acute inflammation model: http://doi.org/10.15490/FAIRDOMHUB.1.MODEL.646.1 .
The model for Fig. 1 is available in live form in JWS-Online as https://jjj.bio.vu.nl/models/?id=abudukelimu.
https://jjj.bio.vu.nl/models/experiments/abudukelimu2019_fig1/simulate will reproduce figure 1. https://jjj.bio.vu.nl/models/?id=abudukelimu is the database entry for the abudukelimu1 model.

The standard model for the chronic mode of inflammation is the same, except that it starts from initial conditions that are equal to the steady state conditions of the acute-mode model at 30 fM/min of CRA influx. Using the standard model all computations can be performed by adjusting a limited number of parameter values, inserting a protease infusion reaction, and altering a limited number of initial states, as specified below.

For the chronic mode computations, Copasi reported numerical issues. These did not affect the results we report however. In an additional set of computations, which essentially reproduced the above mentioned ones, we therefore used a more stable model for the chronic mode in which the fibroblast levels had been fixed to zero. The corresponding model file is called '0 CRA chronic start at SS CRA 30.cps'. Fixing the fibroblast concentrations to zero is not essential for the results, but makes the numerical behavior more robust. In the control analysis calculations for the chronic model, Copasi still reported numerical problems, but these

have to do with the spurious and extremely low levels of MMP7 and MMP8 due to the low levels of the fibroblasts that produce them.

Table S2 shows that steady state and time dependence simulations were in accordance with each other.

TNF and MMP7 concentration upgrade of the models

All computations for the present paper were completed by using the model prepared and tested in Abulikemu et al 2018. Then, little attention was paid to the unit in which concentrations were expressed, except for the concentration of fibroblasts, which we found important for modelling the effect of confluency. This led to a predicted TNF concentration in the acute inflammation of 50 fM, i.e. much lower than levels reported for human serum (approximately 25 (1.0 pM) and 11 pg/mL (0.4 pM) for patients and healthy individuals, respectively (Arican et al., 2005). A posteriori we modulated the standard model so as to obtain a model predicting the concentrations of TNF and MMP7 both to be higher by a factor of 1000 at all times, with all other concentrations being unaffected.

We hereto devised the following more general strategy writing x for TNF and MMP7 and y for the other variables. We assumed that the differential equation for x is a function of x, other variables y, and constants k, κ_i, and K_i:

$$\frac{dx}{dt} = k \cdot \sum_{i=1}^{n} f\left(\frac{x}{K_i}, \frac{y}{L_i}, \kappa_i\right)$$

and

$$\frac{dy}{dt} = l \cdot \sum_{i=1}^{n} g\left(\frac{x}{K_i}, \frac{y}{L_i}, \lambda_i\right)$$

We define: $K_i' \stackrel{\text{def}}{=} \alpha \cdot K_i$ and $k' \stackrel{\text{def}}{=} \alpha \cdot k$ and keep all other parameters the same. Writing x' and y' for the concentration variables x and y in the transformed system, we find:

$$\frac{dx'}{dt} = k' \cdot \sum_{i=1}^{n} f\left(\frac{x'}{K_i'}, y, \kappa_i\right) = \alpha \cdot k \cdot \sum_{i=1}^{n} f\left(\frac{x'/\alpha}{K_i}, \frac{y}{L_i}, \kappa_i\right)$$

and:

$$\frac{dy'}{dt} = l \cdot \sum_{i=1}^{n} g\left(\frac{x'/\alpha}{K_i}, \frac{y'}{L_i}, \lambda_i\right)$$

So that:

$$\frac{d\left(x'/\alpha\right)}{dt} = k \cdot \sum_{i=1}^{n} f\left(\frac{x'/\alpha}{K_i}, \frac{y}{L_i}, \kappa_i\right)$$

Provided that we take $x'(0) = \alpha \cdot x(0)$ and $y'(0) = y(0)$, this means that $x'(t) \equiv \alpha \cdot x(t)$ and $y'(t) \equiv y(t)$, so that variables x are larger by the factor α at all times, whilst variables y are the same.

Following this strategy, we increased the initial value of TNF as well as its synthesis rate constant (see R24), both by the factor 1000, and we decreased the rate constant for the killing of fibroblasts (R16) by the same factor. For MMP7 we increased the initial value and the rate constant of its synthesis (R19), and decreased the rate constant for its catalysis of CRA clip off from healthy fibroblasts (R14), all by the same factor of 1000. We checked that for a CRA influx rate of 16.7, the acute standard model continued to predict the same concentration values, except for a thousand fold increase in TNF and MMP7 levels. By computation with the transformed model, we checked that the predicted TNF level for the chronic inflammation state became 50 pM and that Fig. 4A was unaffected save the change in TNF concentrations.

Sensitivity analysis and control analysis

For the acute mode, Table S3 shows the distribution of control of the various concentrations over all processes in terms of concentration control coefficients. Table S4 does the same for the chronic mode.

(Re-)computation of Figures and Tables

For Figure 1, the file ‘bacterial infection and protease influx’ may be used, setting the protease influx rate to the desired value. This model is the same as the standard acute model except that an irreversible protease influx reaction was added, and the levels of HealthyBacteria and Dying Bacteria were left variable and initialized at 10^{-6} and 0 fM, respectively. The healthy fibroblasts, MMP8 and bacterial levels all exhibited stationary oscillations after some 5 days. For precise reproduction of the figure, the initial state chosen and the interval size chosen matter. For Fig. 1 we used 60 minutes interval size, total simulation time 5760 minutes and initial state as in the Copasi file in the Background folder.

Figure 2 was obtained as described for Tables S3 and S4, below. The file ‘CRA 0 initial SS acute inflammation-altered units’ was used, its CRA influx rate set to 1 fM/min, and control analysis executed to obtain the concentration control coefficients for the acute mode. To obtain the control coefficients for the chronic mode, the CRA influx was set to 30 fM/min, the steady state computed, the initial values stepwise (i.e sequentially) equated to the required steady state values, the CRA influx reset to 1, and the control analysis executed.

To reproduce Figure 3, one may use the file called ‘CRA 0 initial SS acute inflammation.cps’. In ‘Parameter overview’ set the value for the parameter ‘R3_CRAinflux’ to 0.001 fM/min and click ‘commit’. Click ‘Metabolic Control Analysis’ and then ‘Concentration Control Coefficients’ (Or ‘Sensitivities’) and note the value for the control of TNF by R3_CRAinflux (0.00199699). Alternatively click ‘Sensitivities’ to find 0.00199699. Ensure that the scaled coefficients are reported. Then repeat this procedure for higher values of CRA_influx, keeping the initial values of all concentrations the same. This produces the control coefficients of TNF with respect to CRA influx reported in Fig. 3A for the acute mode,

as well as the control coefficients of TNF with respect to TNF production rate (0.998972 for the control coefficient and 0.998971 for the sensitivity). For the chronic mode (Figures 3C and 3D) the same is done but then with the file called '0 CRA chronic start at SS CRA 30.cps'. The difference is that the latter file always starts with initial values of the high inflammation at 30 fM/min CRA influx and has the fibroblast levels fixed to zero in order to generate computational stability.

The data for Figures 4 and 5 are in the excel file HW_Fig4_5. Figure 4A was obtained by increasing the CRA influx rates step-by-step, i.e. by using the standard acute model, running that at CRA influx=0, computing the steady state, setting the initial values to these steady-state values, then setting the CRA influx to the next higher value on the abscissa, i.e. 0.00001, re-computing the steady state, and setting the initial values to the new steady state's values. (Starting each time from the 0 CRA influx initial conditions rather than from the previous steady state as initial conditions, led to a transition to the chronic state already at a CRA influx of 10 fM/min.) Then we changed the initial values of TNF to 100 pM, or 0.1 fM, calculated the subsequent time relaxation, noted the final TNF vale and confirmed that this corresponded to the stable steady state value. Subsequently, the CRA influx was set to the next higher value on the abscissa, and the procedure iterated. Fig 4B was obtained by using the standard acute model, running that at CRA influx=30 fM/min, computing the steady state, set the initial values to these steady state's values, then set the CRA influx to the first value on the abscissa below 30 fM/min, re-compute the steady state, and set the initial values to the new steady state's values, iterating stepwise, then change the initial values of TNF to 100 pM, 10 pM, or 0.1 fM, calculate the subsequent time relaxation, note the final TNF value, and infer that this corresponds to the stable steady-state value. This was iterated stepping down the CRA influx rates.

Figure 5 was obtained following the same stepwise procedure, carrying out steady state analysis and then asking Copasi for the stability analysis, and noting the largest real part of all eigenvalues. This led to the blue and red lines, connecting the computed points (not shown) for the acute and the chronic mode, respectively. The chronic branch was computed down to a CRA influx of 0.00001 and then even 0 fM/min, and then the CRA level was again increased stepwise to obtain the eigenvalues indicated by the blue circles (which are seen to coincide with the points obtained in the CRA-downward computation). We also carried out the CRA downward and back upward computation in a version of the chronic model that had been altered slightly by fixing the Healthy Fibroblasts at zero. Below CRA-influx rates of 0.1 the result diverged from the two other curves, suggesting that the positive eigenvalues observed in the chronic mode below 0.01, were due to an instability in the autocatalytic growth of fibroblasts.

For Figure 6 the data and figures are in the excel file HW_Fig6. The standard acute inflammation Copasi file 'CRA 0 initial SS acute inflammation.cps'. For the acute mode (Fig. 6A) we first set the CRA flux to 0, computed the steady state, set the initial values to these steady state's values, set CRA influx to the value on the abscissa, computed the new steady

state, made the new steady state values the initial values, then changed the Healthy Fibroblast initial value to a 'search value' (second column in the excel file), and computed again the steady state as well as the time dependence. According to the report by Copasi, the final fibroblast level found by the steady state calculation was denoted as stable acute (if high), stable chronic (if low), or as unstable (in between). The solid green, dashed red and solid red lines were drawn to connect the stable acute states, the unstable states and the stable chronic states, respectively. By zooming into initial fibroblast levels around a transition point above which the time dependence resulted in the acute stable steady state value, and below which the time dependence resulted in the stable chronic state value, that transition point was determined. These transition points (called 'wobble points') were connected by the full blue line. For Fig. 6B we did the same except that we always started from the chronic state: we first set the CRA flux to 30 fM/min, computed the steady state, set the initial values to these steady state's values, set CRA influx to the value on the abscissa, computed the new steady state, made the new steady state values the initial values, then changed the Healthy Fibroblast initial value to a 'search value' (second column in the excel file), and computed again the steady state as well as the time dependence. Using to the report by Copasi, the final fibroblast level found by the steady state calculation was denoted as stable acute (if high), stable chronic (if low), or as unstable (in between). The solid green, dashed red and solid red lines were drawn to connect the stable acute states, the unstable states and the stable chronic states, respectively. By zooming into initial fibroblast levels around a transition point, above which the time dependence resulted in the acute stable steady state value and below which the time dependence resulted in the stable chronic state value, that transition point was determined. These transition points (called 'wobble points') were connected by the full blue line. The steady states are equal between Figs A and B, but the wobble lines differ.

Figure 7 we used the Copasi models 'Fibringrowth and acute inflammation.cps' and 'Fibringrowth and chronic inflammation.cps'. These correspond to the standard models for the acute and the chronic mode, respectively, discussed above, except for the addition of a fibroblast ingrowth process Free_space -> HealthyFibr with a first order rate constant and CRA influx rate as defined by the corresponding axes in the figure.

For Figure 8 the steady-state chronic-inflammation level of TNF was computed for CRA influxes of 0.1 fM/min, 0.5 fM/min and 3.0 fM/min, each time starting from the initial state at 30 fM/min and using the standard file for chronic inflammation '0 CRA chronic start at SS CRA 30.cps'. The steady state values obtained were then made initial values, except that of the drug, which was set to an initial value of 10^6 fM and made variable, as was 'FLC_drug' at a value of zero. In (B) fibroblasts were added as well at time zero, to a concentration of 5 fM. In (C) fibroblast influx was started instead, at a fixed rate of 0.01 fM/min. The time evolution of the system was computed by Copasi and the TNF levels attained after 6 hours were calculated and reported by the black circle. If the TNF level subsequently changed with time, the level was annotated by 'us' for 'unstable'. If it did not change with time and the same value was attained after taking the variable values as initial values *and* if performing a steady state

computation with Copasi reported the steady state to be stable, it was annotated with 's'. Further development (if any) in time of the TNF during the subsequent 1 day was also computed for the unstable cases. The blue line was computed as in Fig. 3B and represents the chronic-inflammation steady-states of the model.

For Table 1, one uses the file '0 CRA chronic start at SS CRA 30_altered units.cps' and resets the initial value for TNF and/or for Healthy Fibroblasts of 50 and 0 fM respectively to the values indicated and then runs a steady state analysis to find the final steady state values for both, as evidence for 'cure' or 'no cure'. Information is in the excel file HW_Fig6.

For Table S1, we used the file called 'CRA 0 initial SS acute inflammation.cps', and saved the 'Parameter overview' to file, which we then turned into the excel file 'parameter values acute mode version C'.

For Table S2, we re-used the file used for Fig.1, setting the protease influx to 1 fM/min and running steady state and time dependence, the latter at various resolution settings of Copasi. We also inspected the Jacobian and the eigenvalues in order to decide whether the recurrence of peaks was due to numerical error or real. We found the same times and amplitudes of recurrence, as well as the same minimum level between the first and the second peak of Fig. 1D, independent of Copasi settings. The amplitude at the minimum was not an extremely low number. The time period between the peaks remained constant during much longer simulations, i.e. was not stochastic and the steady state was unstable as judged by a positive real part of one eigenvalue. All of this provides evidence that the recurrent peaks are not due to numerical inaccuracy.

For Table S3 (acute inflammation) and Fig. 2 one may use the file called 'CRA 0 initial SS acute inflammation.cps', in 'Parameter overview' set the value for the parameter 'R3_CRAinflux' to 0, 16.7 or 30.0 fM/min and click 'commit'. Then go to 'Tasks' and to 'Metabolic Control Analysis' (or 'Sensitivities', but note that you want the scaled ones; Results at 16.7 are slightly different from the Metabolic Control Analysis results, due to different computation methodology), click 'Run' and, when results appear 'Concentration Control Coefficients', and round off to one digit after the decimal point. The variable 'washout' is a catalytic parameter always equal to zero; hence the corresponding control coefficients are omitted. Empty space is also omitted as variable as it is always assigned 1000 fM minus the two fibroblast concentrations, in our models. Controls related to Bacteria, and peptide drug were zero and are omitted. Data are stored in the excel file 'HW_SM_Table_3_acute'.

For Table S4 (chronic inflammation) and Fig. 2 please use the file called '0 CRA chronic start at SS CRA 30.cps'. In 'Parameter overview' set the value for the parameter 'R3_CRAinflux' to 0.1 or 1.0 fM/min (A CRA_influx of zero led to an equilibrium steady state, hence without values for the concentration control coefficients) and click 'commit'. Then go to 'Tasks' and to 'Metabolic Control Analysis' (or 'Sensitivities', but note that you want the scaled ones), click 'Run' and, when results appear 'Concentration Control Coefficients', and cut off after 1 digit after the decimal point. In the model file the fibroblast concentrations

were fixed to zero. The control of MMP7 and MMP8 are not reported because their concentrations were always zero due to the zero-ness of the fibroblast concentrations, and neither are the controls by their washouts, by their coupled CRA (BAFF) degradation, nor the controls by processes linked to fibroblasts, peptide drug and bacteria (because these were all zero). Control by CRA binding (Reaction 22) and by FLC binding (reaction 23) was always zero, because these processes are at equilibrium. The variable 'washout' is a catalytic parameter always equal to zero; hence the corresponding control coefficients are omitted. CRA is occasionally called 'BAFF' in the files. Data are stored in the excel file 'HW_SM_Table_4_chronic'.

Numerical aspects

In the computations of the chronic branch of states, a numerical problem had been occurring. The Copasi software reported that it could not compute the steady states. Accordingly, we could not use its stability-analysis facility either. We then calculated the evolution of the system with time and found that it always evolved to the same values for the state variables TNF and fibroblast level (where 'the same' should be interpreted as 'smaller than 10^{-40} in absolute value'). The fibroblast level was extremely close to zero, but of course in a digital computer it is unlikely to arrive at the true zero in a computation. Concluding that the steady state value of fibroblasts should be equal to a true zero, we fixed it to true zero in most of our subsequent computations. We did the same for the dying fibroblasts. This then re-enabled Copasi to compute the steady states, which it reported to be stable, and to calculate their eigenvalues. (To enable Copasi to compute the steady states, it sufficed to set the relevant variable to zero; there was no need to fix them, but for clarity we did). This procedure however, might have compromised our inference that the chronic mode consisted of stable steady states. We therefore took to an alternative method of validating the stability of these steady states: we computed the steady state, took the values of all variables as initial conditions for our subsequent calculations, then perturbed the fibroblast level away from zero, and calculated the subsequent time dependence. For small perturbations of the fibroblast level, we observed that the model returned asymptotically to the zero fibroblast state, making that state asymptotically stable in the sense of Lyapunoff (Westerhoff and Van Dam, 1987; reference 57 of main text), i.e. confirming the stability of that state.

Table S1. Specification of the standard model, set to compute innate inflammation at zero CRA influx rate.

Filename: CRA 0 initial SS acute inflammation:<br>Standard acute case used for generating concentration control analysis in supplementary materials Ablukemi et al				
**Variable Name**	**Type**	**Unit**	**Initial Concentrati on**	
CRA	reactions	fmol/l	0.00999412	
MMP8	reactions	fmol/l	999509	
washout	reactions	fmol/l	1	
FLC	reactions	fmol/l	0.000999412	
MMP7	reactions	fmol/l	99951	
Protease	reactions	fmol/l	1	
Bcells	fixed	fmol/l	1	
drug	fixed	fmol/l	0	
FLC_drug	fixed	fmol/l	0	
DyingFibr	reactions	fmol/l	0	
HealthyFibr	reactions	fmol/l	999.509	
TNFalpha	reactions	fmol/l	0.475162	
HealthyBacteria	fixed	fmol/l	0	
free_space	assignment	fmol/l	0.491	
DyingBacteria	fixed	fmol/l	0	
MastCells_FLC	reactions	fmol/l	9.98E-05	
MastCells_FLC_CRA	reactions	fmol/l	9.98E-07	
MastCells	reactions	fmol/l	0.0998992	
Total space	fixed	fmol/l	1000	
Process and Parameter				
**Process (unit)**	**Reaction**	**Rate Law**	**Reactions Parameters (unit)**	**Value**
R1_CRA_degradation fmol/(l*min)	CRA + MMP8 -> MMP8	Mass action (irreversible)	k1 l/(min*fmol)	0.0001
R2_CRA_washout fmol/(l*min)	CRA + washout -> washout	Mass action (irreversible)	k1 l/(min*fmol)	0.01

R3_CRAinflux fmol/(l*min)	-> CRA	Constant flux (irreversible)	v fmol/(min*l)	0
R4_FLC_washout fmol/(l*min)	FLC + washout -> washout	Mass action (irreversible)	k1 l/(min*fmol)	0.01
R5_MMP7_washout fmol/(l*min)	MMP7 + washout -> washout	Mass action (irreversible)	k1 l/(min*fmol)	0.01
R6_MMP8_washout fmol/(l*min)	MMP8 + washout -> washout	Mass action (irreversible)	k1 l/(min*fmol)	0.01
R7_Protease_washout fmol/(l*min)	Protease + washout -> washout	Mass action (irreversible)	k1 l/(min*fmol)	0.01

R8_TNFalpha_washout fmol/(l*min)	TNFalpha + washout -> washout	Mass action (irreversible)	k1 l/(min*fmol)	0.01
R9_FLC_production fmol/(l*min)	CRA + Bcells -> FLC + CRA + Bcells	Rate Law for B_FLC_production	k1 l/(min*fmol)	0.001
R10_drug_washout fmol/(l*min)	drug + washout -> washout	Mass action (irreversible)	k1 l/(min*fmol)	0.0001
R11_FLC_drug_binding fmol/(l*min)	drug + FLC = FLC_drug	Mass action (reversible)	k1 l/(min*fmol)	1.00E-05
			k2 1/min	1.00 E-05
R12_FLC_drug_washout fmol/(l*min)	FLC_drug + washout -> washout	Mass action (irreversible)	k1 l/(min*fmol)	0.00 01
R13_CRA_Secretion_DyingFibr fmol/(l*min)	DyingFibr -> CRA	Mass action (irreversible)	k1 1/min	0.00 1
R14_CRAClipOffHealthyFibr fmol/(l*min)	HealthyFibr + MMP7 -> CRA + MMP7 + HealthyFibr	Rate Law for BAFF clipoff	k1 l/(min*fmol)	1.00 E-08
R15_DyingFibroblast_death fmol/(l*min)	DyingFibr ->	Mass action (irreversible)	k1 1/min	0.2
R16_Healthy_to_Dying_fibroblast fmol/(l*min)	HealthyFibr + TNFalpha -> DyingFibr + TNFalpha	Mass action (irreversible)	k1 l/(min*fmol)	5.00E-07
R17_HealthyBacteriaProduction fmol/(l*min)	HealthyBacteria -> 2 * HealthyBacteria	Mass action (irreversible)	k1 1/min	0.01
R18_HealthyFibProduction fmol/(l*min)	free_space + HealthyFibr -> 2 * HealthyFibr	Mass action (irreversible)	k1 l/(min*fmol)	4.85E-07

R19_MMP7_release_Healthy Fibr fmol/(l*min)	HealthyFibr -> HealthyFibr + MMP7	Mass action (irreversible)	k1 1/min	1
R20_MMP8_release_Healthy Fibr fmol/(l*min)	HealthyFibr -> HealthyFibr + 100 * MMP8	Mass action (irreversible)	k1 1/min	0.1
R21_Healthy_to_Dying_Bact eria fmol/(l*min)	HealthyBacteria + Protease -> DyingBacteria + Protease	Mass action (irreversible)	k1 l/(min*fmol)	0.005
R22_CRA_binding fmol/(l*min)	MastCells_FLC + CRA = MastCells_FLC_C RA	Mass action (reversible)	k1 l/(min*fmol)	0.1
			k2 1/min	0.1
R23_FLC_binding fmol/(l*min)	FLC + MastCells = MastCells_FLC	Mass action (reversible)	k1 l/(min*fmol)	0.1
			k2 1/min	0.1
R24_TNFalpha_producti on fmol/(l*min)	MastCells_FLC_C RA -> TNFalpha + MastCells_FLC_C RA	Mass action (irreversible)	k1 1/min	

R25_Protease_production fmol/(l*min)	MastCells_FLC_C RA -> Protease + MastCells_FLC_C RA	Mass action (irreversible)	k1 1/min	5000
R26_DyingBacteria secrete CRA fmol/(l*min)	DyingBacteria -> 1000 * CRA	Mass action (irreversible)	k1 1/min	65
R27_Dyingbacteria die fmol/(l*min)	DyingBacteria ->	Mass action (irreversible)	k1 1/min	10

Table S1. Specification of the standard model, set to compute innate inflammation at zero CRA influx rate. This model is encoded by the Copasi file ‘CRA 0 initial SS acute inflammation-B.cps’ (the addition –B is only relevant for the model naming) and an XML file by the same name. This Table is also in the excel file ‘Parameter values acute mode version C-altered’. The phrase ‘altered’ refers to the differences with the model in Abulikemu et al 2018, which are highlighted in yellow and lead to improved magnitudes of the TNF and MMP7 levels as discussed above.

Table S2. Concentration of various species at steady state and at 4 days into the time course simulation.

Species	Concentration in time course (4 days)	Concentration at Steady-state
**Healthy Bacteria**	$<10^{-18}$	$<10^{-18}$
**Dying Bacteria**	$<10^{-18}$	$<10^{-18}$
**TNF**	0.50	0.50
**Healthy Fibroblasts**	999.5	999.5
**Dying Fibroblasts**	0.0012	0.0013
**CRA**	0.010	0.010
**MMP8**	$999\ 10^3$	$999\ 10^3$
**MMP7**	$99.9\ 10^3$	$99.9\ 10^3$
**IgE**	0.0010	0.0010
**Protease**	100	100
**Mast cells**	0.100	0.100

Table S2. Concentration of various species at steady state and at 4 days into the time course simulation (fM, mostly rounded to two most significant digits). Protease influx rate was set at 1.0 fM/min.

Table S3. Sensitivity coefficients for the acute inflammation mode (included as separate supplementary file: datasheet 3)

Concentration control coefficients for acute-branch inflammation at three CRA influx values											
J_CRA=0, acute	<--Controlled variable-->										
Controlling parameter ↓	CRA	FLC	thyFibroblasts	DyingFibroblasts	Protease	MMP7	MMP8	TNF	MastCells_FLC	MastCells_FLC_CRA	MastCells
R1_CRA_degradation	-1,0	-1,0	0,0	-2,0	-2,0	0,0	0,0	-2,0	-1,0	-2,0	0,0
R2_CRA_washout	0,0	0,0	0,0	0,0	0,0	0,0	0,0	0,0	0,0	0,0	0,0
R3_CRAinflux	0,0	0,0	0,0	0,0	0,0	0,0	0,0	0,0	0,0	0,0	0,0
R4_FLC_washout	0,0	-1,0	0,0	-1,0	-1,0	0,0	0,0	-1,0	-1,0	-1,0	0,0
R5_MMP7_washout	-1,0	-1,0	0,0	-2,0	-2,0	-1,0	0,0	-2,0	-1,0	-2,0	0,0
R6_MMP8_washout	1,0	1,0	0,0	2,0	2,0	0,0	-1,0	2,0	1,0	2,0	0,0
R7_Protease_washout	0,0	0,0	0,0	0,0	-1,0	0,0	0,0	0,0	0,0	0,0	0,0
R8_TNFalpha_washout	0,0	0,0	0,0	-1,0	0,0	0,0	0,0	-1,0	0,0	0,0	0,0
R9_FLC_production	0,0	1,0	0,0	1,0	1,0	0,0	0,0	1,0	1,0	1,0	0,0
R10_drug_washout	0,0	0,0	0,0	0,0	0,0	0,0	0,0	0,0	0,0	0,0	0,0
R11_FLC_drug_binding	0,0	0,0	0,0	0,0	0,0	0,0	0,0	0,0	0,0	0,0	0,0
R12_FLC_drug_washout	0,0	0,0	0,0	0,0	0,0	0,0	0,0	0,0	0,0	0,0	0,0
R13_CRA_Secretion_DyingFibr	0,0	0,0	0,0	0,0	0,0	0,0	0,0	0,0	0,0	0,0	0,0
R14_CRAClipOffHealthyFibr	1,0	1,0	0,0	2,0	2,0	0,0	0,0	2,0	1,0	2,0	0,0
R15_DyingFibroblast_death	0,0	0,0	0,0	-1,0	0,0	0,0	0,0	0,0	0,0	0,0	0,0
R16_Healthy_to_Dying_fibroblast	0,0	0,0	0,0	1,0	0,0	0,0	0,0	0,0	0,0	0,0	0,0
R17_HealthyBacteriaProduction	0,0	0,0	0,0	0,0	0,0	0,0	0,0	0,0	0,0	0,0	0,0
R18_HealthyFibProduction	0,0	0,0	0,0	0,0	0,0	0,0	0,0	0,0	0,0	0,0	0,0
R19_MMP7_release_HealthyFibr	1,0	1,0	0,0	2,0	2,0	1,0	0,0	2,0	1,0	2,0	0,0
R20_MMP8_release_HealthyFibr	-1,0	-1,0	0,0	-2,0	-2,0	0,0	1,0	-2,0	-1,0	-2,0	0,0
R21_Healthy_to_Dying_Bacteria	0,0	0,0	0,0	0,0	0,0	0,0	0,0	0,0	0,0	0,0	0,0
R22_CRA_binding	0,0	0,0	0,0	0,0	0,0	0,0	0,0	0,0	0,0	0,0	0,0
R23_FLC_binding	0,0	0,0	0,0	0,0	0,0	0,0	0,0	0,0	0,0	0,0	0,0
R24_TNFalpha_production	0,0	0,0	0,0	1,0	0,0	0,0	0,0	1,0	0,0	0,0	0,0
R25_Protease_production	0,0	0,0	0,0	0,0	1,0	0,0	0,0	0,0	0,0	0,0	0,0
R26_DyingBacteria secrete CRA	0,0	0,0	0,0	0,0	0,0	0,0	0,0	0,0	0,0	0,0	0,0
R27_Dyingbacteria die	0,0	0,0	0,0	0,0	0,0	0,0	0,0	0,0	0,0	0,0	0,0
Sum over parameters	0,0	0,0	0,0	0,0	0,0	0,0	0,0	0,0	0,0	0,0	0,0
J_CRA=16.7 acute	CRA	FLC	HealthyFibr	DyingFibr	Protease	MMP7	MMP8	TNFalpha	MastCells_FLC	MastCells_FLC_CRA	MastCells
R1_CRA_degradation	-6,2	-6,2	5,5	-6,7	-12,1	5,5	5,5	-12,1	-6,0	-12,1	0,2
R2_CRA_washout	0,0	0,0	0,0	0,0	0,0	0,0	0,0	0,0	0,0	0,0	0,0
R3_CRAinflux	6,0	6,0	-5,3	6,5	11,8	-5,3	-5,3	11,8	5,8	11,8	-0,2
R4_FLC_washout	-2,6	-3,6	2,7	-3,3	-6,0	2,7	2,7	-6,0	-3,4	-6,0	0,1
R5_MMP7_washout	-0,2	-0,2	0,2	-0,2	-0,3	-0,8	0,2	-0,3	-0,2	-0,3	0,0
R6_MMP8_washout	6,2	6,2	-5,5	6,7	12,1	-5,5	-6,5	12,1	6,0	12,1	-0,2
R7_Protease_washout	0,0	0,0	0,0	0,0	-1,0	0,0	0,0	0,0	0,0	0,0	0,0
R8_TNFalpha_washout	-2,6	-2,6	2,8	-3,4	-5,2	2,8	2,8	-6,2	-2,5	-5,2	0,1
R9_FLC_production	2,6	3,6	-2,7	3,3	6,0	-2,7	-2,7	6,0	3,4	6,0	-0,1
R10_drug_washout	0,0	0,0	0,0	0,0	0,0	0,0	0,0	0,0	0,0	0,0	0,0
R11_FLC_drug_binding	0,0	0,0	0,0	0,0	0,0	0,0	0,0	0,0	0,0	0,0	0,0
R12_FLC_drug_washout	0,0	0,0	0,0	0,0	0,0	0,0	0,0	0,0	0,0	0,0	0,0
R13_CRA_Secretion_DyingFibr	0,0	0,0	0,0	0,0	0,0	0,0	0,0	0,0	0,0	0,0	0,0

R14_CRAClipOffHealthyFibr	0,2	0,2	-0,2	0,2	0,3	-0,2	-0,2	0,3	0,2	0,3	0,0
R15_DyingFibroblast_death	0,0	0,0	0,0	-1,0	0,0	0,0	0,0	0,0	0,0	0,0	0,0
R16_Healthy_to_Dying_fibroblast	2,6	2,6	-2,8	3,4	5,2	-2,8	-2,8	5,2	2,5	5,2	-0,1
R17_HealthyBacteriaProduction	0,0	0,0	0,0	0,0	0,0	0,0	0,0	0,0	0,0	0,0	0,0
R18_HealthyFibProduction	-2,6	-2,6	2,8	-2,4	-5,2	2,8	2,8	-5,2	-2,5	-5,2	0,1
R19_MMP7_release_HealthyFibr	0,2	0,2	-0,2	0,2	0,3	0,8	-0,2	0,3	0,2	0,3	0,0
R20_MMP8_release_HealthyFibr	-6,2	-6,2	5,5	-6,7	-12,1	5,5	6,5	-12,1	-6,0	-12,1	0,2
R21_Healthy_to_Dying_Bacteria	0,0	0,0	0,0	0,0	0,0	0,0	0,0	0,0	0,0	0,0	0,0
R22_CRA_binding	0,0	0,0	0,0	0,0	0,0	0,0	0,0	0,0	0,0	0,0	0,0
R23_FLC_binding	0,0	0,0	0,0	0,0	0,0	0,0	0,0	0,0	0,0	0,0	0,0
R24_TNFalpha_production	2,6	2,6	-2,8	3,4	5,2	-2,8	-2,8	6,2	2,5	5,2	-0,1
R25_Protease_production	0,0	0,0	0,0	0,0	1,0	0,0	0,0	0,0	0,0	0,0	0,0
R26_DyingBacteria secrete CRA	0,0	0,0	0,0	0,0	0,0	0,0	0,0	0,0	0,0	0,0	0,0
R27_Dyingbacteria die	0,0	0,0	0,0	0,0	0,0	0,0	0,0	0,0	0,0	0,0	0,0
Sum	0,0	0,0	0,0	0,0	0,0	0,0	0,0	0,0	0,0	0,0	0,0
CRA_influx=30, acute	CRA	FLC	HealthyFibr	DyingFibr	Protease	MMP7	MMP8	TNFalpha	MastCells_FLC	MastCells_FLC_CRA	MastCells
R1_CRA_degradation	0,0	0,0	inf	inf	0,0	inf	inf	0,0	0,0	0,0	0,0
R2_CRA_washout	-1,0	-1,0	inf	inf	0,0	inf	inf	0,0	1,0	0,0	2,0
R3_CRAinflux	1,0	1,0	inf	inf	0,0	inf	inf	0,0	-1,0	0,0	-2,0
R4_FLC_washout	0,0	-1,0	inf	inf	0,0	inf	inf	0,0	0,0	0,0	1,0
R5_MMP7_washout	0,0	0,0	inf	inf	0,0	inf	inf	0,0	0,0	0,0	0,0
R6_MMP8_washout	0,0	0,0	inf	inf	0,0	inf	inf	0,0	0,0	0,0	0,0
R7_Protease_washout	0,0	0,0	inf	inf	-1,0	inf	inf	0,0	0,0	0,0	0,0
R8_TNFalpha_washout	0,0	0,0	inf	inf	0,0	inf	inf	-1,0	0,0	0,0	0,0
R9_FLC_production	0,0	1,0	inf	inf	0,0	inf	inf	0,0	0,0	0,0	-1,0
R10_drug_washout	0,0	0,0	inf	inf	0,0	inf	inf	0,0	0,0	0,0	0,0
R11_FLC_drug_binding	0,0	0,0	inf	inf	0,0	inf	inf	0,0	0,0	0,0	0,0
R12_FLC_drug_washout	0,0	0,0	inf	inf	0,0	inf	inf	0,0	0,0	0,0	0,0
R13_CRA_Secretion_DyingFibr	0,0	0,0	inf	inf	0,0	inf	inf	0,0	0,0	0,0	0,0
R14_CRAClipOffHealthyFibr	0,0	0,0	inf	inf	0,0	inf	inf	0,0	0,0	0,0	0,0
R15_DyingFibroblast_death	0,0	0,0	inf	inf	0,0	inf	inf	0,0	0,0	0,0	0,0
R16_Healthy_to_Dying_fibroblast	0,0	0,0	inf	inf	0,0	inf	inf	0,0	0,0	0,0	0,0
R17_HealthyBacteriaProduction	0,0	0,0	inf	inf	0,0	inf	inf	0,0	0,0	0,0	0,0
R18_HealthyFibProduction	0,0	0,0	inf	inf	0,0	inf	inf	0,0	0,0	0,0	0,0
R19_MMP7_release_HealthyFibr	0,0	0,0	inf	inf	0,0	inf	inf	0,0	0,0	0,0	0,0
R20_MMP8_release_HealthyFibr	0,0	0,0	inf	inf	0,0	inf	inf	0,0	0,0	0,0	0,0
R21_Healthy_to_Dying_Bacteria	0,0	0,0	inf	inf	0,0	inf	inf	0,0	0,0	0,0	0,0
R22_CRA_binding	0,0	0,0	inf	inf	0,0	inf	inf	0,0	0,0	0,0	0,0
R23_FLC_binding	0,0	0,0	inf	inf	0,0	inf	inf	0,0	0,0	0,0	0,0
R24_TNFalpha_production	0,0	0,0	inf	inf	0,0	inf	inf	1,0	0,0	0,0	0,0
R25_Protease_production	0,0	0,0	inf	inf	1,0	inf	inf	0,0	0,0	0,0	0,0
R26_DyingBacteria secrete CRA	0,0	0,0	inf	inf	0,0	inf	inf	0,0	0,0	0,0	0,0
R27_Dyingbacteria die	0,0	0,0	inf	inf	0,0	inf	inf	0,0	0,0	0,0	0,0
Sum	0,0	0,0	inf	inf	0,0	inf	inf	0,0	0,0	0,0	0,0

Table S3 (in separate datasheet). Sensitivity coefficients for the acute inflammation mode at CRA influx rates of 0, 16.7 and 30.0 fM/min. inf' refers to a value reported as infinity, minus infinity or 'not a number'. 'Sum' refers to the sum of the control coefficients over all the processes, above it.

Table S4. Nonzero/non-infinite control coefficients of the chronic inflammation mode (also included as separate supplementary file: datasheet 4)

Concentration control coefficients for chronic inflammation at three CRA influx rates							
Controlling parameter ↓	<--Controlled concentration-->						
CRA_influx=0.1, chronic mode	CRA	FLC	Protease	TNF	Mast Cells_FLC	Mast Cells_FLC_CRA	Mast Cells
R2_CRA_washout	-1,0	-1,0	-0,3	-0,3	0,8	-0,3	1,8
R3_CRAinflux	1,0	1,0	0,3	0,3	-0,8	0,3	-1,8
R4_FLC_washout	0,0	-1,0	-0,1	-0,1	-0,1	-0,1	0,9
R7_Protease_washout	0,0	0,0	-1,0	0,0	0,0	0,0	0,0
R8_TNF_washout	0,0	0,0	0,0	-1,0	0,0	0,0	0,0
R9_FLC_production	0,0	1,0	0,1	0,1	0,1	0,1	-0,9
R24_TNF_production	0,0	0,0	0,0	1,0	0,0	0,0	0,0
R25_Protease_production	0,0	0,0	1,0	0,0	0,0	0,0	0,0
sum	0,0	0,0	0,0	0,0	0,0	0,0	0,0
**CRA_influx=1, chronic mode**	**CRA**	**FLC**	**Protease**	**TNF**	**Mast Cells_FLC**	**Mast Cells_FLC_CRA**	**Mast Cells**
R2_CRA_washout	-1,0	-1,0	0,0	0,0	1,0	0,0	2,0
R3_CRAinflux	1,0	1,0	0,0	0,0	-1,0	0,0	-2,0
R4_FLC_washout	0,0	-1,0	0,0	0,0	0,0	0,0	1,0
R7_Protease_washout	0,0	0,0	-1,0	0,0	0,0	0,0	0,0
R8_TNF_washout	0,0	0,0	0,0	-1,0	0,0	0,0	0,0
R9_FLC_production	0,0	1,0	0,0	0,0	0,0	0,0	-1,0
R24_TNF_production	0,0	0,0	0,0	1,0	0,0	0,0	0,0
R25_Protease_production	0,0	0,0	1,0	0,0	0,0	0,0	0,0
sum	0,0	0,0	0,0	0,0	0,0	0,0	0,0
**CRA influx =16.7, chronic mode**	**CRA**	**FLC**	**Protease**	**TNF**	**Mast Cells_FLC**	**Mast Cells_FLC_CRA**	**Mast Cells**
R2_CRA_washout	-1,0	-1,0	0,0	0,0	1,0	0,0	2,0
R3_CRAinflux	1,0	1,0	0,0	0,0	-1,0	0,0	-2,0
R4_FLC_washout	0,0	-1,0	0,0	0,0	0,0	0,0	1,0
R7_Protease_washout	0,0	0,0	-1,0	0,0	0,0	0,0	0,0
R8_TNF_washout	0,0	0,0	0,0	-1,0	0,0	0,0	0,0
R9_FLC_production	0,0	1,0	0,0	0,0	0,0	0,0	-1,0
R24_TNF_production	0,0	0,0	0,0	1,0	0,0	0,0	0,0
R25_Protease_production	0,0	0,0	1,0	0,0	0,0	0,0	0,0
sum	0,0	0,0	0,0	0,0	0,0	0,0	0,0

Table S4. (in separate datasheet) Nonzero/non-infinite control coefficients of the chronic inflammation mode at three CRA influx rates. CRA influx rates were 0.1, 1.0 and 16.7 fM/min. Fibroblasts were fixed to zero. 'sum' refers to the sum of the coefficients for any concentration over all 15 reaction rate constants and should equal zero according to the summation law of Metabolic Control Analysis (Westerhoff, H.V et al., 2009; main text ref 51).
