# Supplementary material for: Complex Stability and an Irrevertible Transition Reverted by Peptide and Fibroblasts in a Dynamic Model of Innate Immunity
Source: Front Immunol. 2020 Feb 14;10:3091. doi: 10.3389/fimmu.2019.03091 (PMC7033641; doi:10.3389/fimmu.2019.03091)
Supplement: Data Sheet 2 — The Copasi and SBML files, as well as the Copasi software used. Live model version is available in JWS-Online through https://jjj.bio.vu.nl/models/?id=abudukelimu. [file Data_Sheet_2.zip › NewBackground to Abulikemu et al 2020-20200123T085315Z-001/Background to Abulikemu et al 2020/Figure 1/RS_New_Fig_1_Bacterial infection.pptx]

## Slide 1
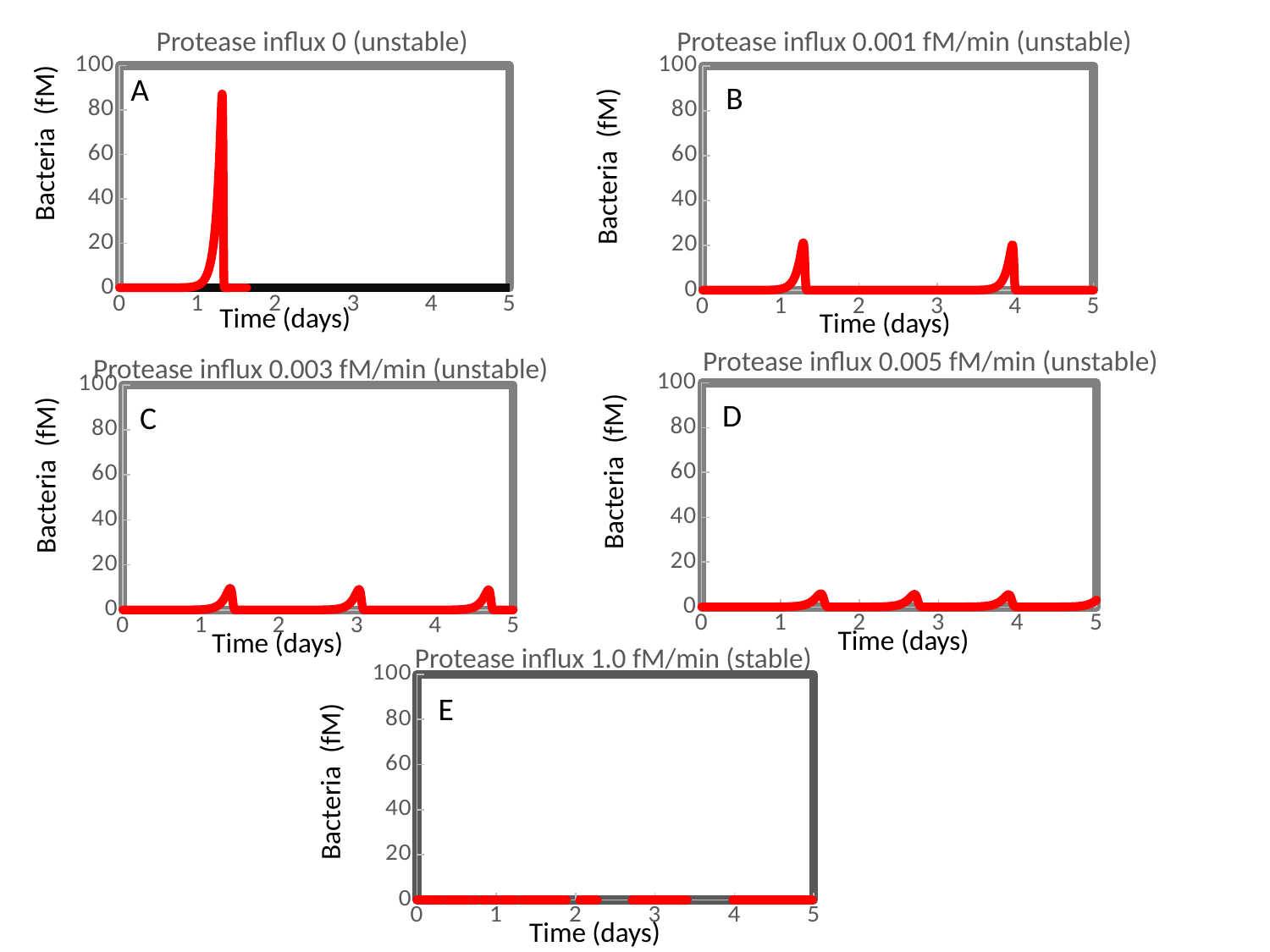

Protease influx 0 (unstable)
### Chart
| Category | |
|---|---|Bacteria (fM)
Time (days)
A
Protease influx 0.001 fM/min (unstable)
### Chart
| Category | |
|---|---|Bacteria (fM)
Time (days)
B
Protease influx 0.005 fM/min (unstable)
### Chart
| Category | |
|---|---|Bacteria (fM)
Time (days)
D
Protease influx 0.003 fM/min (unstable)
### Chart
| Category | [Bacteria] |
|---|---|Bacteria (fM)
Time (days)
C
Protease influx 1.0 fM/min (stable)
### Chart
| Category | |
|---|---|Bacteria (fM)
Time (days)
E
