# Supplementary material for: Complex Stability and an Irrevertible Transition Reverted by Peptide and Fibroblasts in a Dynamic Model of Innate Immunity
Source: Front Immunol. 2020 Feb 14;10:3091. doi: 10.3389/fimmu.2019.03091 (PMC7033641; doi:10.3389/fimmu.2019.03091)
Supplement: Data Sheet 2 — The Copasi and SBML files, as well as the Copasi software used. Live model version is available in JWS-Online through https://jjj.bio.vu.nl/models/?id=abudukelimu. [file Data_Sheet_2.zip › NewBackground to Abulikemu et al 2020-20200123T085315Z-001/Background to Abulikemu et al 2020/Figure7_8/NEW Figure 7_New Heatmap and drug effects.pptx]

## Slide 1
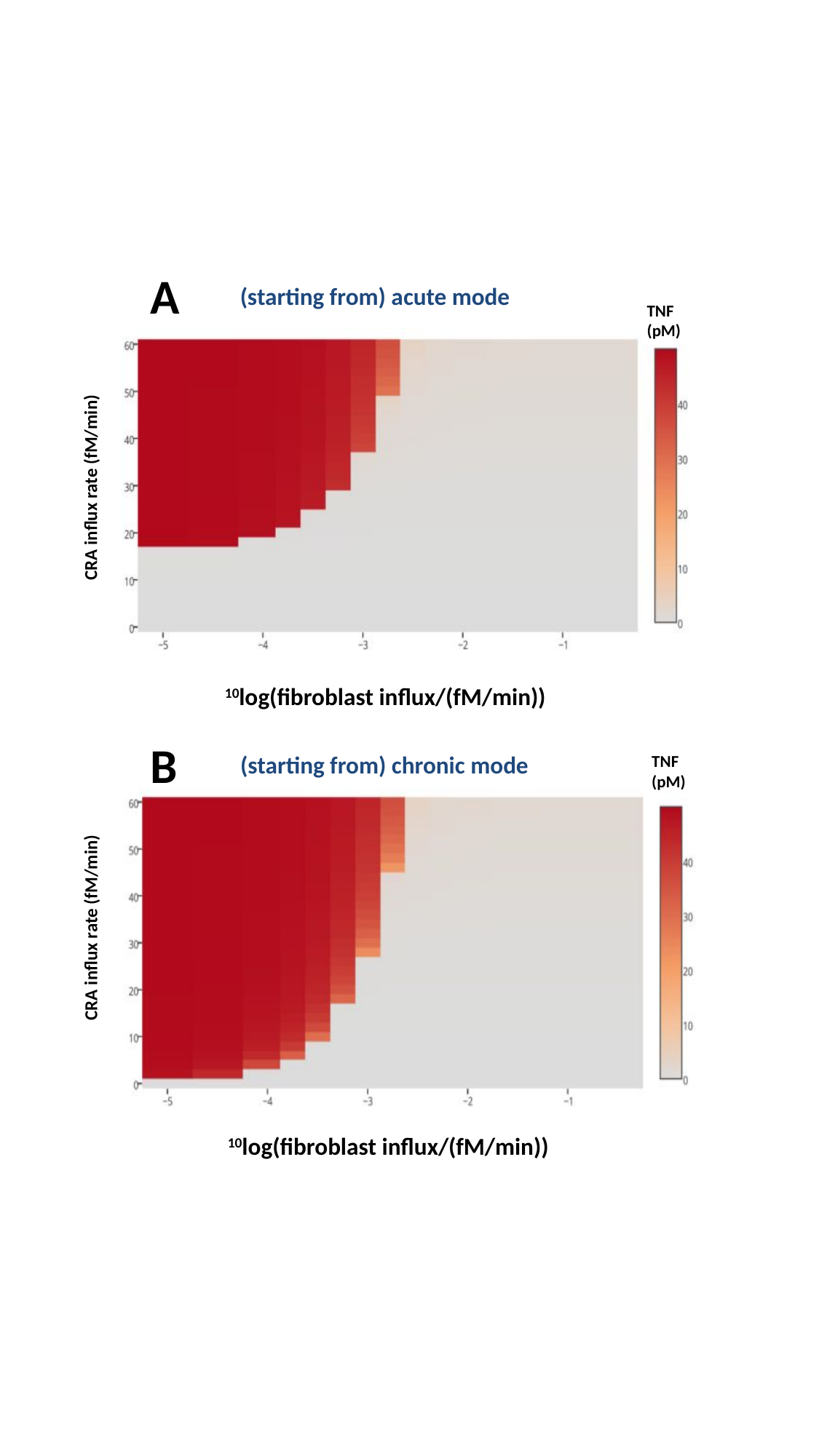

A
TNF (pM)
(starting from) acute mode
 CRA influx rate (fM/min)
10log(fibroblast influx/(fM/min))
B
TNF (pM)
 CRA influx rate (fM/min)
10log(fibroblast influx/(fM/min))
(starting from) chronic mode
