# Supplementary figures and images for: Complex Stability and an Irrevertible Transition Reverted by Peptide and Fibroblasts in a Dynamic Model of Innate Immunity
Source: Front Immunol. 2020 Feb 14;10:3091. doi: 10.3389/fimmu.2019.03091 (PMC7033641; doi:10.3389/fimmu.2019.03091)

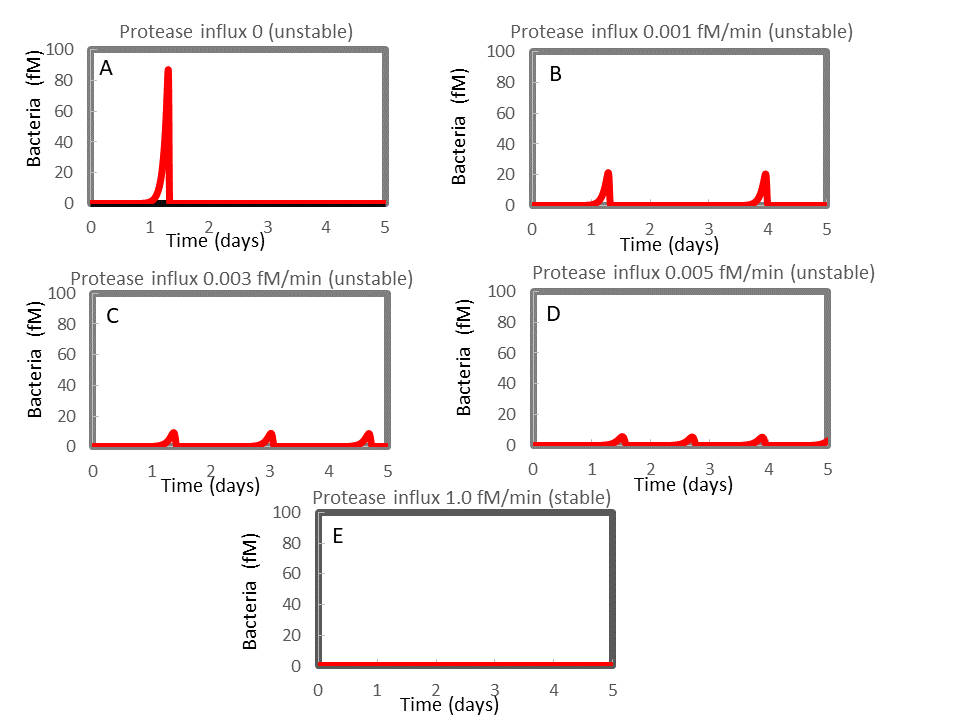

Supplement: Data Sheet 2 — The Copasi and SBML files, as well as the Copasi software used. Live model version is available in JWS-Online through https://jjj.bio.vu.nl/models/?id=abudukelimu. [file Data_Sheet_2.zip › NewBackground to Abulikemu et al 2020-20200123T085315Z-001/Background to Abulikemu et al 2020/Figure 1/RS_New_Fig_1_Bacterial infection.png]

## Slide 1
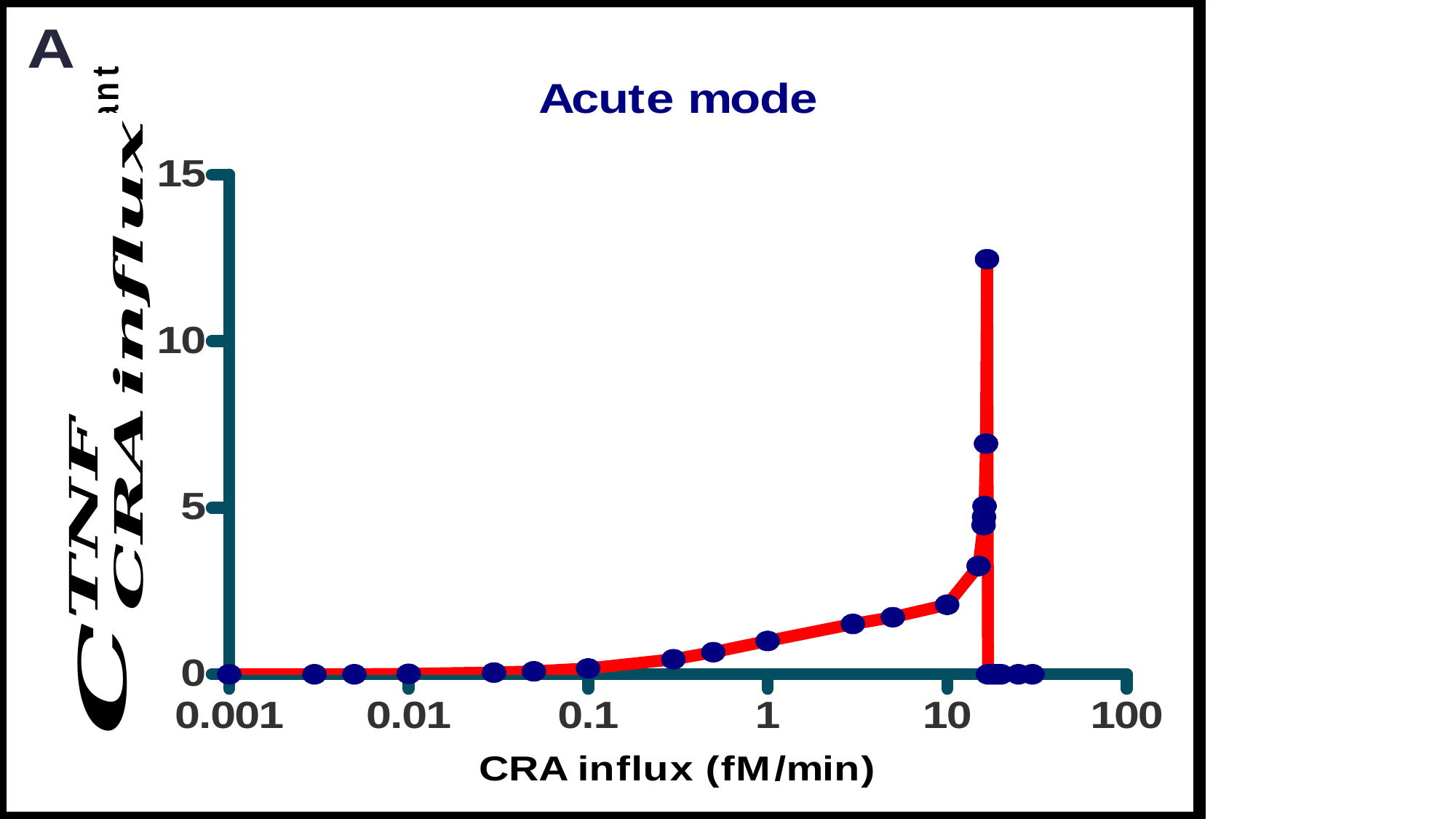

## Slide 2
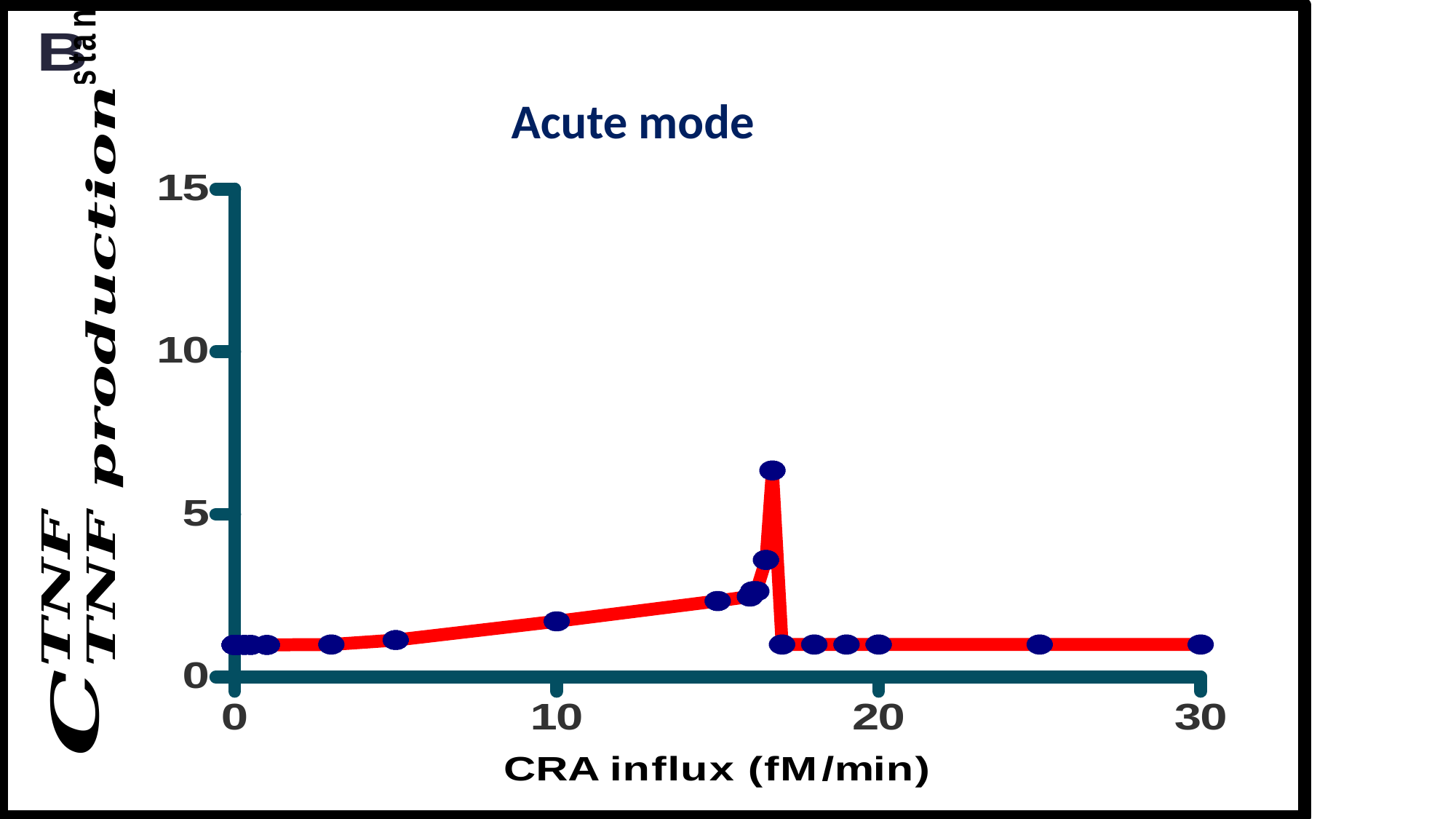

Acute mode

## Slide 3
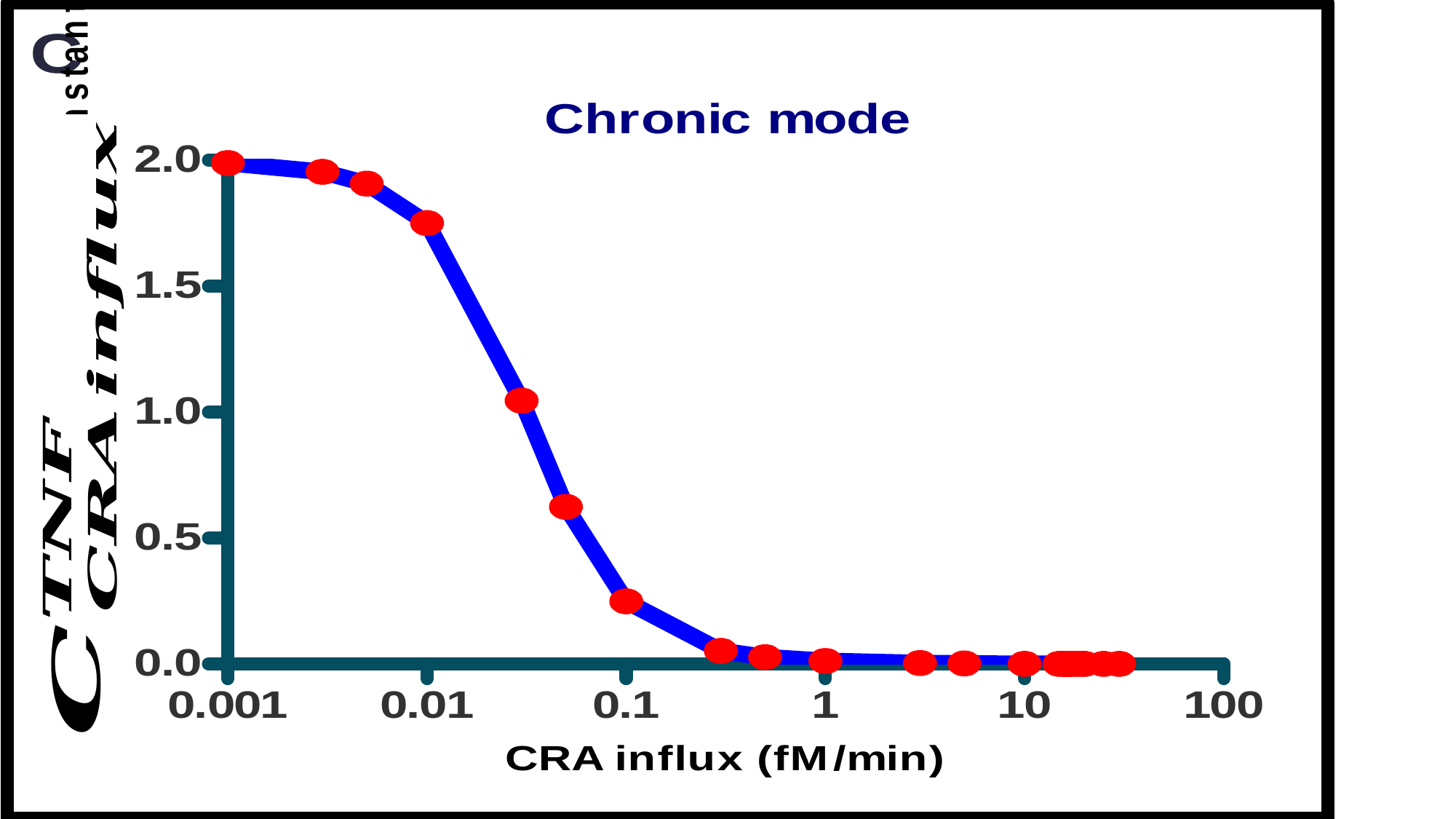

## Slide 4
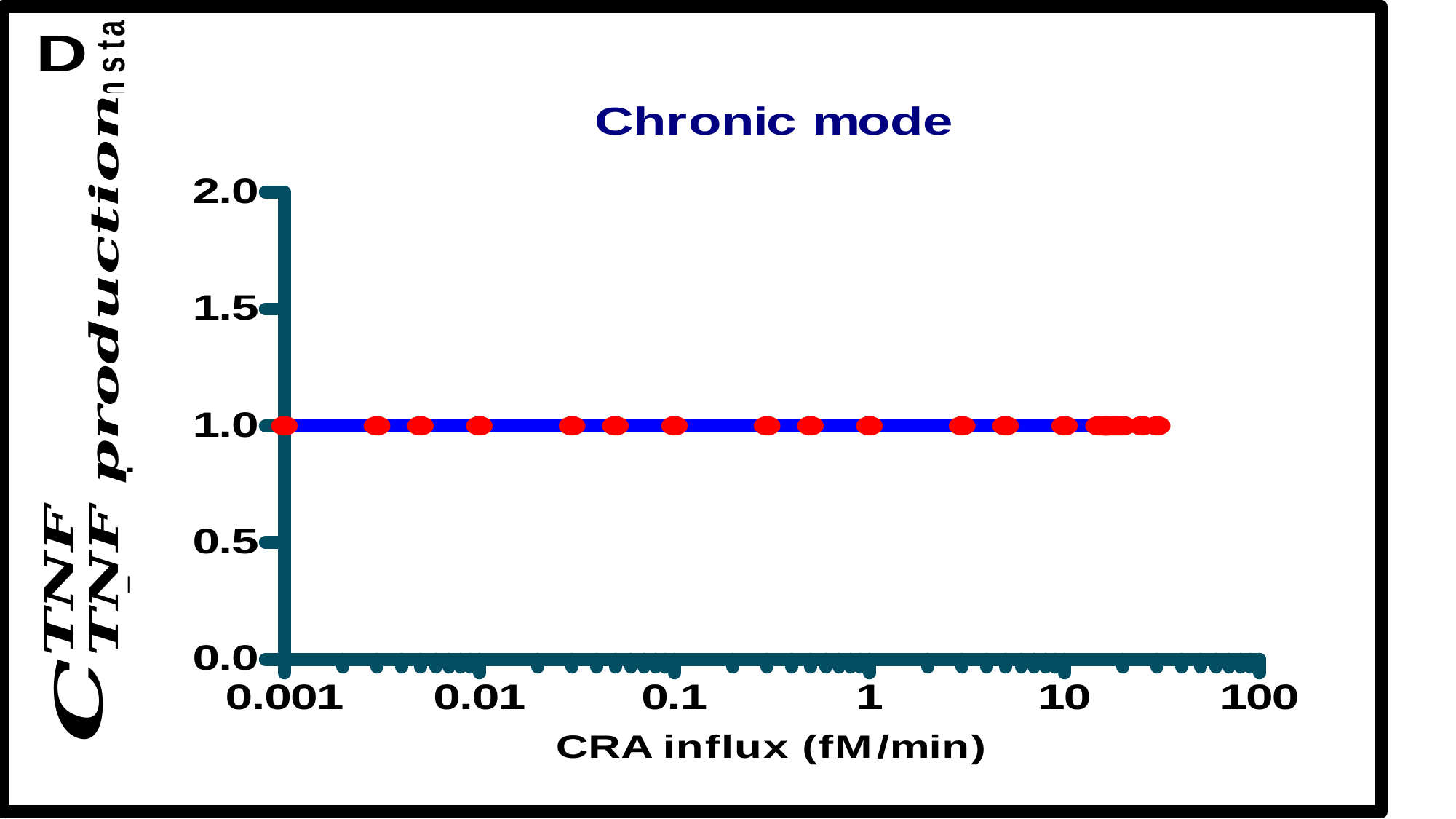

Supplement: Data Sheet 2 — The Copasi and SBML files, as well as the Copasi software used. Live model version is available in JWS-Online through https://jjj.bio.vu.nl/models/?id=abudukelimu. [file Data_Sheet_2.zip › NewBackground to Abulikemu et al 2020-20200123T085315Z-001/Background to Abulikemu et al 2020/Figure 3/Fig_3.pptx]
