# Supplementary material for: Complex Stability and an Irrevertible Transition Reverted by Peptide and Fibroblasts in a Dynamic Model of Innate Immunity
Source: Front Immunol. 2020 Feb 14;10:3091. doi: 10.3389/fimmu.2019.03091 (PMC7033641; doi:10.3389/fimmu.2019.03091)
Supplement: Data Sheet 3 — Supplementary Table S3. [file Data_Sheet_3.pdf]

| Concentration control coefficients for acute-branch inflammation at three CRA influx values | | | | | | | | | | | |
|---|---|---|---|---|---|---|---|---|---|---|---|
| J_CRA=0, acute | <--Controlled variable--> | | | | | | | | | | |
| Controlling parameter ↓ | CRA | FLC | thyFibroblasts | DyingFibroblasts | Protease | MMP7 | MMP8 | TNF | MastCells_FLC | MastCells_FLC_CRA | MastCells |
| R1_CRA_degradation | -1.0 | -1.0 | 0.0 | -2.0 | -2.0 | 0.0 | 0.0 | -2.0 | -1.0 | -2.0 | 0.0 |
| R2_CRA_washout | 0.0 | 0.0 | 0.0 | 0.0 | 0.0 | 0.0 | 0.0 | 0.0 | 0.0 | 0.0 | 0.0 |
| R3_CRAinflux | 0.0 | 0.0 | 0.0 | 0.0 | 0.0 | 0.0 | 0.0 | 0.0 | 0.0 | 0.0 | 0.0 |
| R4_FLC_washout | 0.0 | -1.0 | 0.0 | -1.0 | -1.0 | 0.0 | 0.0 | -1.0 | -1.0 | -1.0 | 0.0 |
| R5_MMP7_washout | -1.0 | -1.0 | 0.0 | -2.0 | -2.0 | -1.0 | 0.0 | -2.0 | -1.0 | -2.0 | 0.0 |
| R6_MMP8_washout | 1.0 | 1.0 | 0.0 | 2.0 | 2.0 | 0.0 | -1.0 | 2.0 | 1.0 | 2.0 | 0.0 |
| R7_Protease_washout | 0.0 | 0.0 | 0.0 | 0.0 | -1.0 | 0.0 | 0.0 | 0.0 | 0.0 | 0.0 | 0.0 |
| R8_TNFalpha_washout | 0.0 | 0.0 | 0.0 | -1.0 | 0.0 | 0.0 | 0.0 | -1.0 | 0.0 | 0.0 | 0.0 |
| R9_FLC_production | 0.0 | 1.0 | 0.0 | 1.0 | 1.0 | 0.0 | 0.0 | 1.0 | 1.0 | 1.0 | 0.0 |
| R10_drug_washout | 0.0 | 0.0 | 0.0 | 0.0 | 0.0 | 0.0 | 0.0 | 0.0 | 0.0 | 0.0 | 0.0 |
| R11_FLC_drug_binding | 0.0 | 0.0 | 0.0 | 0.0 | 0.0 | 0.0 | 0.0 | 0.0 | 0.0 | 0.0 | 0.0 |
| R12_FLC_drug_washout | 0.0 | 0.0 | 0.0 | 0.0 | 0.0 | 0.0 | 0.0 | 0.0 | 0.0 | 0.0 | 0.0 |
| R13_CRA_Secretion_DyingFibr | 0.0 | 0.0 | 0.0 | 0.0 | 0.0 | 0.0 | 0.0 | 0.0 | 0.0 | 0.0 | 0.0 |
| R14_CRAClipOffHealthyFibr | 1.0 | 1.0 | 0.0 | 2.0 | 2.0 | 0.0 | 0.0 | 2.0 | 1.0 | 2.0 | 0.0 |
| R15_DyingFibroblast_death | 0.0 | 0.0 | 0.0 | -1.0 | 0.0 | 0.0 | 0.0 | 0.0 | 0.0 | 0.0 | 0.0 |
| R16_Healthy_to_Dying_fibroblast | 0.0 | 0.0 | 0.0 | 1.0 | 0.0 | 0.0 | 0.0 | 0.0 | 0.0 | 0.0 | 0.0 |
| R17_HealthyBacteriaProduction | 0.0 | 0.0 | 0.0 | 0.0 | 0.0 | 0.0 | 0.0 | 0.0 | 0.0 | 0.0 | 0.0 |
| R18_HealthyFibProduction | 0.0 | 0.0 | 0.0 | 0.0 | 0.0 | 0.0 | 0.0 | 0.0 | 0.0 | 0.0 | 0.0 |
| R19_MMP7_release_HealthyFibr | 1.0 | 1.0 | 0.0 | 2.0 | 2.0 | 1.0 | 0.0 | 2.0 | 1.0 | 2.0 | 0.0 |
| R20_MMP8_release_HealthyFibr | -1.0 | -1.0 | 0.0 | -2.0 | -2.0 | 0.0 | 1.0 | -2.0 | -1.0 | -2.0 | 0.0 |
| R21_Healthy_to_Dying_Bacteria | 0.0 | 0.0 | 0.0 | 0.0 | 0.0 | 0.0 | 0.0 | 0.0 | 0.0 | 0.0 | 0.0 |
| R22_CRA_binding | 0.0 | 0.0 | 0.0 | 0.0 | 0.0 | 0.0 | 0.0 | 0.0 | 0.0 | 0.0 | 0.0 |
| R23_FLC_binding | 0.0 | 0.0 | 0.0 | 0.0 | 0.0 | 0.0 | 0.0 | 0.0 | 0.0 | 0.0 | 0.0 |
| R24_TNFalpha_production | 0.0 | 0.0 | 0.0 | 1.0 | 0.0 | 0.0 | 0.0 | 1.0 | 0.0 | 0.0 | 0.0 |
| R25_Protease_production | 0.0 | 0.0 | 0.0 | 0.0 | 1.0 | 0.0 | 0.0 | 0.0 | 0.0 | 0.0 | 0.0 |
| R26_DyingBacteria secrete CRA | 0.0 | 0.0 | 0.0 | 0.0 | 0.0 | 0.0 | 0.0 | 0.0 | 0.0 | 0.0 | 0.0 |
| R27_Dyingbacteria die | 0.0 | 0.0 | 0.0 | 0.0 | 0.0 | 0.0 | 0.0 | 0.0 | 0.0 | 0.0 | 0.0 |
| Sum over parameters | 0.0 | 0.0 | 0.0 | 0.0 | 0.0 | 0.0 | 0.0 | 0.0 | 0.0 | 0.0 | 0.0 |
| | | | | | | | | | | | |
| J_CRA=16.7 acute | CRA | FLC | HealthyFibr | DyingFibr | Protease | MMP7 | MMP8 | TNFalpha | MastCells_FLC | MastCells_FLC_CRA | MastCells |
| R1_CRA_degradation | -6.2 | -6.2 | 5.5 | -6.7 | -12.1 | 5.5 | 5.5 | -12.1 | -6.0 | -12.1 | 0.2 |
| R2_CRA_washout | 0.0 | 0.0 | 0.0 | 0.0 | 0.0 | 0.0 | 0.0 | 0.0 | 0.0 | 0.0 | 0.0 |
| R3_CRAinflux | 6.0 | 6.0 | -5.3 | 6.5 | 11.8 | -5.3 | -5.3 | 11.8 | 5.8 | 11.8 | -0.2 |
| R4_FLC_washout | -2.6 | -3.6 | 2.7 | -3.3 | -6.0 | 2.7 | 2.7 | -6.0 | -3.4 | -6.0 | 0.1 |
| R5_MMP7_washout | -0.2 | -0.2 | 0.2 | -0.2 | -0.3 | -0.8 | 0.2 | -0.3 | -0.2 | -0.3 | 0.0 |
| R6_MMP8_washout | 6.2 | 6.2 | -5.5 | 6.7 | 12.1 | -5.5 | -6.5 | 12.1 | 6.0 | 12.1 | -0.2 |
| R7_Protease_washout | 0.0 | 0.0 | 0.0 | 0.0 | -1.0 | 0.0 | 0.0 | 0.0 | 0.0 | 0.0 | 0.0 |
| R8_TNFalpha_washout | -2.6 | -2.6 | 2.8 | -3.4 | -5.2 | 2.8 | 2.8 | -6.2 | -2.5 | -5.2 | 0.1 |
| R9_FLC_production | 2.6 | 3.6 | -2.7 | 3.3 | 6.0 | -2.7 | -2.7 | 6.0 | 3.4 | 6.0 | -0.1 |
| R10_drug_washout | 0.0 | 0.0 | 0.0 | 0.0 | 0.0 | 0.0 | 0.0 | 0.0 | 0.0 | 0.0 | 0.0 |
| R11_FLC_drug_binding | 0.0 | 0.0 | 0.0 | 0.0 | 0.0 | 0.0 | 0.0 | 0.0 | 0.0 | 0.0 | 0.0 |
| R12_FLC_drug_washout | 0.0 | 0.0 | 0.0 | 0.0 | 0.0 | 0.0 | 0.0 | 0.0 | 0.0 | 0.0 | 0.0 |
| R13_CRA_Secretion_DyingFibr | 0.0 | 0.0 | 0.0 | 0.0 | 0.0 | 0.0 | 0.0 | 0.0 | 0.0 | 0.0 | 0.0 |

| | | | | | | | | | | | |
|---|---|---|---|---|---|---|---|---|---|---|---|
| **R14_CRAClipOffHealthyFibr** | 0.2 | 0.2 | -0.2 | 0.2 | 0.3 | -0.2 | -0.2 | 0.3 | 0.2 | 0.3 | 0.0 |
| **R15_DyingFibroblast_death** | 0.0 | 0.0 | 0.0 | -1.0 | 0.0 | 0.0 | 0.0 | 0.0 | 0.0 | 0.0 | 0.0 |
| **R16_Healthy_to_Dying_fibroblast** | 2.6 | 2.6 | -2.8 | 3.4 | 5.2 | -2.8 | -2.8 | 5.2 | 2.5 | 5.2 | -0.1 |
| **R17_HealthyBacteriaProduction** | 0.0 | 0.0 | 0.0 | 0.0 | 0.0 | 0.0 | 0.0 | 0.0 | 0.0 | 0.0 | 0.0 |
| **R18_HealthyFibProduction** | -2.6 | -2.6 | 2.8 | -2.4 | -5.2 | 2.8 | 2.8 | -5.2 | -2.5 | -5.2 | 0.1 |
| **R19_MMP7_release_HealthyFibr** | 0.2 | 0.2 | -0.2 | 0.2 | 0.3 | 0.8 | -0.2 | 0.3 | 0.2 | 0.3 | 0.0 |
| **R20_MMP8_release_HealthyFibr** | -6.2 | -6.2 | 5.5 | -6.7 | -12.1 | 5.5 | 6.5 | -12.1 | -6.0 | -12.1 | 0.2 |
| **R21_Healthy_to_Dying_Bacteria** | 0.0 | 0.0 | 0.0 | 0.0 | 0.0 | 0.0 | 0.0 | 0.0 | 0.0 | 0.0 | 0.0 |
| **R22_CRA_binding** | 0.0 | 0.0 | 0.0 | 0.0 | 0.0 | 0.0 | 0.0 | 0.0 | 0.0 | 0.0 | 0.0 |
| **R23_FLC_binding** | 0.0 | 0.0 | 0.0 | 0.0 | 0.0 | 0.0 | 0.0 | 0.0 | 0.0 | 0.0 | 0.0 |
| **R24_TNFalpha_production** | 2.6 | 2.6 | -2.8 | 3.4 | 5.2 | -2.8 | -2.8 | 6.2 | 2.5 | 5.2 | -0.1 |
| **R25_Protease_production** | 0.0 | 0.0 | 0.0 | 0.0 | 1.0 | 0.0 | 0.0 | 0.0 | 0.0 | 0.0 | 0.0 |
| **R26_DyingBacteria secrete CRA** | 0.0 | 0.0 | 0.0 | 0.0 | 0.0 | 0.0 | 0.0 | 0.0 | 0.0 | 0.0 | 0.0 |
| **R27_Dyingbacteria die** | 0.0 | 0.0 | 0.0 | 0.0 | 0.0 | 0.0 | 0.0 | 0.0 | 0.0 | 0.0 | 0.0 |
| **Sum** | 0.0 | 0.0 | 0.0 | 0.0 | 0.0 | 0.0 | 0.0 | 0.0 | 0.0 | 0.0 | 0.0 |
| | | | | | | | | | | | |
| **CRA_influx=30, acute** | **CRA** | **FLC** | **HealthyFibr** | **DyingFibr** | **Protease** | **MMP7** | **MMP8** | **TNFalpha** | **MastCells_FLC** | **MastCells_FLC_CRA** | **MastCells** |
| **R1_CRA_degradation** | 0.0 | 0.0 | inf | inf | 0.0 | inf | inf | 0.0 | 0.0 | 0.0 | 0.0 |
| **R2_CRA_washout** | -1.0 | -1.0 | inf | inf | 0.0 | inf | inf | 0.0 | 1.0 | 0.0 | 2.0 |
| **R3_CRAinflux** | 1.0 | 1.0 | inf | inf | 0.0 | inf | inf | 0.0 | -1.0 | 0.0 | -2.0 |
| **R4_FLC_washout** | 0.0 | -1.0 | inf | inf | 0.0 | inf | inf | 0.0 | 0.0 | 0.0 | 1.0 |
| **R5_MMP7_washout** | 0.0 | 0.0 | inf | inf | 0.0 | inf | inf | 0.0 | 0.0 | 0.0 | 0.0 |
| **R6_MMP8_washout** | 0.0 | 0.0 | inf | inf | 0.0 | inf | inf | 0.0 | 0.0 | 0.0 | 0.0 |
| **R7_Protease_washout** | 0.0 | 0.0 | inf | inf | -1.0 | inf | inf | 0.0 | 0.0 | 0.0 | 0.0 |
| **R8_TNFalpha_washout** | 0.0 | 0.0 | inf | inf | 0.0 | inf | inf | -1.0 | 0.0 | 0.0 | 0.0 |
| **R9_FLC_production** | 0.0 | 1.0 | inf | inf | 0.0 | inf | inf | 0.0 | 0.0 | 0.0 | -1.0 |
| **R10_drug_washout** | 0.0 | 0.0 | inf | inf | 0.0 | inf | inf | 0.0 | 0.0 | 0.0 | 0.0 |
| **R11_FLC_drug_binding** | 0.0 | 0.0 | inf | inf | 0.0 | inf | inf | 0.0 | 0.0 | 0.0 | 0.0 |
| **R12_FLC_drug_washout** | 0.0 | 0.0 | inf | inf | 0.0 | inf | inf | 0.0 | 0.0 | 0.0 | 0.0 |
| **R13_CRA_Secretion_DyingFibr** | 0.0 | 0.0 | inf | inf | 0.0 | inf | inf | 0.0 | 0.0 | 0.0 | 0.0 |
| **R14_CRAClipOffHealthyFibr** | 0.0 | 0.0 | inf | inf | 0.0 | inf | inf | 0.0 | 0.0 | 0.0 | 0.0 |
| **R15_DyingFibroblast_death** | 0.0 | 0.0 | inf | inf | 0.0 | inf | inf | 0.0 | 0.0 | 0.0 | 0.0 |
| **R16_Healthy_to_Dying_fibroblast** | 0.0 | 0.0 | inf | inf | 0.0 | inf | inf | 0.0 | 0.0 | 0.0 | 0.0 |
| **R17_HealthyBacteriaProduction** | 0.0 | 0.0 | inf | inf | 0.0 | inf | inf | 0.0 | 0.0 | 0.0 | 0.0 |
| **R18_HealthyFibProduction** | 0.0 | 0.0 | inf | inf | 0.0 | inf | inf | 0.0 | 0.0 | 0.0 | 0.0 |
| **R19_MMP7_release_HealthyFibr** | 0.0 | 0.0 | inf | inf | 0.0 | inf | inf | 0.0 | 0.0 | 0.0 | 0.0 |
| **R20_MMP8_release_HealthyFibr** | 0.0 | 0.0 | inf | inf | 0.0 | inf | inf | 0.0 | 0.0 | 0.0 | 0.0 |
| **R21_Healthy_to_Dying_Bacteria** | 0.0 | 0.0 | inf | inf | 0.0 | inf | inf | 0.0 | 0.0 | 0.0 | 0.0 |
| **R22_CRA_binding** | 0.0 | 0.0 | inf | inf | 0.0 | inf | inf | 0.0 | 0.0 | 0.0 | 0.0 |
| **R23_FLC_binding** | 0.0 | 0.0 | inf | inf | 0.0 | inf | inf | 0.0 | 0.0 | 0.0 | 0.0 |
| **R24_TNFalpha_production** | 0.0 | 0.0 | inf | inf | 0.0 | inf | inf | 1.0 | 0.0 | 0.0 | 0.0 |
| **R25_Protease_production** | 0.0 | 0.0 | inf | inf | 1.0 | inf | inf | 0.0 | 0.0 | 0.0 | 0.0 |
| **R26_DyingBacteria secrete CRA** | 0.0 | 0.0 | inf | inf | 0.0 | inf | inf | 0.0 | 0.0 | 0.0 | 0.0 |
| **R27_Dyingbacteria die** | 0.0 | 0.0 | inf | inf | 0.0 | inf | inf | 0.0 | 0.0 | 0.0 | 0.0 |
| **Sum** | 0.0 | 0.0 | inf | inf | 0.0 | inf | inf | 0.0 | 0.0 | 0.0 | 0.0 |

Table S3. Sensitivity coefficients for the acute inflammation mode at CRA influx rates of 0, 16.7 and 30.0 fM/min. inf’ refers to a value reported as infinity, minus infinity or ‘not a number’. ‘Sum’ refers to the sum of the control coefficients over all the processes, above it.
