# Supplementary material for: Complex Stability and an Irrevertible Transition Reverted by Peptide and Fibroblasts in a Dynamic Model of Innate Immunity
Source: Front Immunol. 2020 Feb 14;10:3091. doi: 10.3389/fimmu.2019.03091 (PMC7033641; doi:10.3389/fimmu.2019.03091)
Supplement: Data Sheet 4 — Supplementary Table S4. [file Data_Sheet_4.pdf]

Concentration control coefficients for chronic inflammation at three CRA influx rates							
Controlling parameter ↓	<--Controlled concentration-->						
CRA_influx=0.1, chronic mode	CRA	FLC	Protease	TNF	Mast Cells_FLC	Mast Cells_FLC_CRA	Mast Cells
R2_CRA_washout	-1.0	-1.0	-0.3	-0.3	0.8	-0.3	1.8
R3_CRAinflux	1.0	1.0	0.3	0.3	-0.8	0.3	-1.8
R4_FLC_washout	0.0	-1.0	-0.1	-0.1	-0.1	-0.1	0.9
R7_Protease_washout	0.0	0.0	-1.0	0.0	0.0	0.0	0.0
R8_TNF_washout	0.0	0.0	0.0	-1.0	0.0	0.0	0.0
R9_FLC_production	0.0	1.0	0.1	0.1	0.1	0.1	-0.9
R24_TNF_production	0.0	0.0	0.0	1.0	0.0	0.0	0.0
R25_Protease_production	0.0	0.0	1.0	0.0	0.0	0.0	0.0
sum	0.0	0.0	0.0	0.0	0.0	0.0	0.0
**CRA_influx=1, chronic mode**	**CRA**	**FLC**	**Protease**	**TNF**	**Mast Cells_FLC**	**Mast Cells_FLC_CRA**	**Mast Cells**
R2_CRA_washout	-1.0	-1.0	0.0	0.0	1.0	0.0	2.0
R3_CRAinflux	1.0	1.0	0.0	0.0	-1.0	0.0	-2.0
R4_FLC_washout	0.0	-1.0	0.0	0.0	0.0	0.0	1.0
R7_Protease_washout	0.0	0.0	-1.0	0.0	0.0	0.0	0.0
R8_TNF_washout	0.0	0.0	0.0	-1.0	0.0	0.0	0.0
R9_FLC_production	0.0	1.0	0.0	0.0	0.0	0.0	-1.0
R24_TNF_production	0.0	0.0	0.0	1.0	0.0	0.0	0.0
R25_Protease_production	0.0	0.0	1.0	0.0	0.0	0.0	0.0
sum	0.0	0.0	0.0	0.0	0.0	0.0	0.0
**CRA influx =16.7, chronic mode**	**CRA**	**FLC**	**Protease**	**TNF**	**Mast Cells_FLC**	**Mast Cells_FLC_CRA**	**Mast Cells**
R2_CRA_washout	-1.0	-1.0	0.0	0.0	1.0	0.0	2.0
R3_CRAinflux	1.0	1.0	0.0	0.0	-1.0	0.0	-2.0
R4_FLC_washout	0.0	-1.0	0.0	0.0	0.0	0.0	1.0
R7_Protease_washout	0.0	0.0	-1.0	0.0	0.0	0.0	0.0
R8_TNF_washout	0.0	0.0	0.0	-1.0	0.0	0.0	0.0
R9_FLC_production	0.0	1.0	0.0	0.0	0.0	0.0	-1.0
R24_TNF_production	0.0	0.0	0.0	1.0	0.0	0.0	0.0
R25_Protease_production	0.0	0.0	1.0	0.0	0.0	0.0	0.0
sum	0.0	0.0	0.0	0.0	0.0	0.0	0.0

Table S4. Nonzero/non-infinite control coefficients of the chronic inflammation mode at three CRA influx rates. CRA influx rates were 0.1, 1.0 and 16.7 fM/min. Fibroblasts were fixed to zero. ‘sum’ refers to the sum of the coefficients for any concentration over all 15 reaction rate constants and should equal zero according to the summation law of Metabolic Control Analysis (Westerhoff, H.V et al., 2009; main text ref 51).
